# p53's choice of myocardial death or survival: Oxygen protects infarct myocardium by recruiting p53 on NOS3 promoter through regulation of p53-Lys^118^ acetylation

**DOI:** 10.1002/emmm.201202055

**Published:** 2013-10-01

**Authors:** Rajan Gogna, Esha Madan, Mahmood Khan, Uttam Pati, Periannan Kuppusamy

**Affiliations:** 1 Dorothy M. Davis Heart and Lung Research Institute, Division of Cardiovascular Medicine, Department of Internal Medicine, Ohio State University Wexner Medical CenterColumbus, OH, USA; 2 Department of Emergency Medicine, Ohio State University Wexner Medical CenterColumbus, OH, USA; 3 Transcription and Human Biology Laboratory, School of Biotechnology, Jawaharlal Nehru UniversityNew Delhi, India; 4 Department of Radiology, Geisel School of Medicine at Dartmouth, Dartmouth CollegeHanover, NH, USA

**Keywords:** lysine acetylation, myocardial infarction, NOS3, oxygenation, p53

## Abstract

Myocardial infarction, an irreversible cardiac tissue damage, involves progressive loss of cardiomyocytes due to p53-mediated apoptosis. Oxygenation is known to promote cardiac survival through activation of NOS3 gene. We hypothesized a dual role for p53, which, depending on oxygenation, can elicit apoptotic death signals or NOS3-mediated survival signals in the infarct heart. p53 exhibited a differential DNA-binding, namely, BAX-p53RE in the infarct heart or NOS3-p53RE in the oxygenated heart, which was regulated by oxygen-induced, post-translational modification of p53. In the infarct heart, p53 was heavily acetylated at Lys^118^ residue, which was exclusively reversed in the oxygenated heart, apparently regulated by oxygen-dependent expression of TIP60. The inhibition of Lys^118^ acetylation promoted the generation of NOS3-promoting prosurvival form of p53. Thus, oxygenation switches p53-DNA interaction by regulating p53 core-domain acetylation, promoting a prosurvival transcription activity of p53. Understanding this novel oxygen-p53 survival pathway will open new avenues in cardioprotection molecular therapy.

## INTRODUCTION

Cardiovascular diseases claim more lives than any other disease in the world. Of all forms of cardiovascular diseases, myocardial infarction (MI) accounts for more than 40% of deaths. MI is largely attributed to the permanent loss of cardiomyocytes due to necrotic and apoptotic process of cell death (Kajstura et al, 1996; Yaoita et al, 2000). Studies using animal models of MI indicate that myocyte death due to necrosis is an early event that begins with prolonged ischemia, further exacerbated with the onset of reperfusion, and may last up to 24 h (Eefting et al, 2004; Oerlemans et al, 2012). On the other hand, apoptotic cell death is largely initiated during reperfusion and continues to occur in the MI heart for longer duration (Gottlieb et al, 1994; Zhao et al, 2000). If left untreated, MI will continue to undergo progressive loss of viable cardiomyocytes due to p53-mediated apoptosis, extensive remodelling and deterioration of cardiac function which eventually may lead to congestive heart failure. Thus, there is an increasing need to develop clinically applicable therapies to inhibit the progression of cardiac damage and/or cardiac tissue regeneration in MI patients (Bolli et al, 2011; Laflamme & Murry, 2011). Administration of oxygen cycling to a rat model of MI has been shown to protect cardiomyocytes from ischemia/reflow-induced death and is believed to function through upregulation of NOS3 expression (Cabigas et al, 2006). Recently, we observed that daily administration of oxygen (oxygen-cycling; OxCy; 90 min/day for 4 weeks) to rats with experimentally-induced MI resulted in a significant reduction of infarction and improvement of cardiac function (Khan et al, 2012). The oxygen-cycling also improved engraftment of mesenchymal stem cells (MSC) transplanted in the infarcted myocardium. It was further observed that endothelial nitric oxide synthase (eNOS or NOS3) was overexpressed both in the oxygenated and stem cell-treated hearts (Khan et al, 2012). p53 is an established apoptotic effector in infarct heart (Bialik et al, 1997; Crow et al, 2004). p53 and NOS3 have been shown to have clinical correlation (Alvarado-Vasquez et al, 2007). p53 transcriptionally regulates other members of NOS family as well (Chen et al, 2003). However, if p53 and NOS3 have a relation at the transcriptional level then p53 might have a dual role in the enforcement of ‘death’ or ‘survival’ in the infarct heart. p53 regulates nexus of many cellular pathways, it integrates abnormal signals and in response, induces arrest, apoptosis or DNA-repair in a context-dependent manner (Ko & Prives, 1996; Levine et al, 2006). However, whether p53 might have a role in cell-survival (Gogna et al, 2012a; Madan et al, 2011; Vousden, 2006) and thus possesses the ability to support cardiac survival in oxygenated infarct myocardium is not established. The hypothesis is supported by the evidence that p53 positively regulates the expression of genes whose products are directly involved in evoking anti-apoptotic effects in cancer cells (Janicke et al, 2008). This list of genes includes glutathione peroxidase (Hussain et al, 2004; Tan et al, 1999; Yan & Chen, 2006), manganese superoxide dismutase (Hussain et al, 2004), aldehyde dehydrogenase 4 (Donald et al, 2001), p53-induced glycolysis and apoptosis regulator (TIGAR; Bensaad et al, 2006; Madan et al, 2012), as well as PA26 and Hi95 that encode two proteins of the sestrin family, namely sestrin 1, sestrin 2 (Budanov et al, 2002; Masutani et al, 2005; Velasco-Miguel et al, 1999) and Slug (Wu et al, 2005). Another transcription factor induced by p53 is Krüppel-like factor 4 (Zhang et al, 2000), which induces cell-cycle arrest at the G1/S and G2/M transition (Rowland et al, 2005), thus participates in the cell-survival program. Similarly, Cop1 (constitutively photomorphogenic 1) and Pirh2 (p53-induced protein with a Ring-H2 domain) proteins (Dornan et al, 2004; Fuchs et al, 1998; Leng et al, 2003), p53-induced R2 homolog gene with a p53-binding sequence in intron 1 (Kimura et al, 2003; Monte et al, 2003; Tanaka et al, 2000; Utrera et al, 1998), hematopoietic zinc finger (Braithwaite et al, 2006; Vousden, 2006), heparin-binding epidermal growth factor-like growth factor (Fang et al, 2001), discoidin domain receptor 1 (Ongusaha et al, 2003) and cyclooxygenase 2 (Han et al, 2002) have been shown to be involved in the p53-mediated survival program of cancer cells.

In this study, we observed that the pro-apoptotic p53 gene was overexpressed in the oxygenated-infarct hearts with survival potential. We hypothesized that p53 senses oxygen-induced molecular changes and plays a dual role in generating apoptotic signals in the infarct heart and NOS3-mediated survival signals in the oxygenated heart. We used a rat model of MI induced by permanent ligation of left-anterior-descending (LAD) coronary artery. Rats were exposed to oxygen-cycling 90 min/day for 4 weeks (Khan et al, 2012). Heart tissues harvested from the infarct region were used for analysis. The results showed that p53 exhibits a differential DNA binding, switching from BAX-p53RE in the infarct heart to NOS3-p53RE in the oxygenated heart, apparently regulated by oxygen-dependent TIP60 acetylase expression and post-translational modification of p53 core domain at p53-Lys^118^ residue. The study establishes a new role of p53 in cardioprotection.

## RESULTS

### p53 is upregulated in both apoptotic MI hearts and surviving oxygenated MI hearts

**Figure 1:**
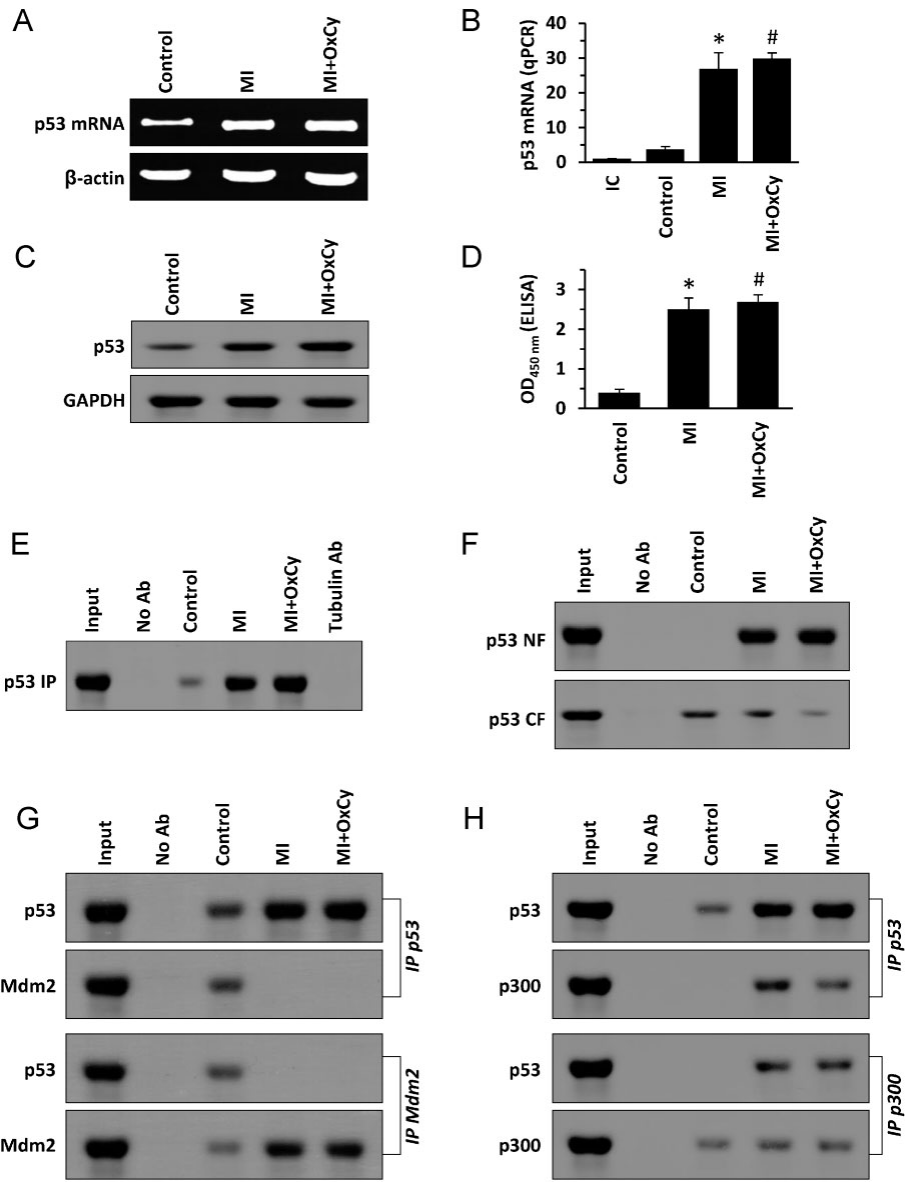
p53 is upregulated and transcriptionally active in both infarct and oxygenated hearts RT-PCR images show that p53 mRNA is upregulated in both MI and MI + OxCy hearts.Real-time qPCR values, expressed relative to internal control (IC), show significantly higher levels of p53 mRNA in both MI and MI + OxCy hearts when compared to control. Data represent mean ± SD of five independent measurements. *p = 4.3E−06; ^#^*p* = 1.5E−09.Western-blot images show that p53 protein is increased in both MI and MI + OxCy hearts.*In vivo* ELISA results show significantly higher levels of p53 in both MI and MI + OxCy hearts when compared to control. Data represent mean ± SD of five independent measurements. *p = 2.0E−06; ^#^*p* = 5.2E−09.Immunoprecipitation (IP) data show increase in p53 protein level in MI and MI + OxCy hearts.Subcellular localization data of p53, obtained by IP of nuclear (NF) and cytoplasmic (CF) fractions from MI and MI + OxCy heart tissues, show that p53 is present in the nuclear fraction of both MI and MI + OxCy hearts.Binding of p53 to its inhibitor Mdm2 was analysed using co-IP with both anti-p53 and anti-Mdm2 antibodies. The data show that p53 is bound to Mdm2 only in the control hearts, while the MI and MI + OxCy hearts show no interaction of p53 with Mdm2.p53 transcriptional activity was probed in MI and MI + OxCy hearts by analysing the binding of p53 to its transcriptional activator p300 using co-immunoprecipitation with both anti-p53 and anti-Mdm2 antibodies. The data show that p53 is bound to p300 in both MI and MI + OxCy hearts. Overall, the results established that p53 is upregulated, nuclear, stabilized, and transcriptionally potent in the infarct and oxygenated hearts.

MI was induced in rats by an ischemia-reperfusion protocol as described previously (Khan et al, 2012). Briefly, following thoracotomy, LAD coronary artery was occluded for 60 min and released subsequently to induce ischemia-reflow injury. After 2 days of recovery period, the MI rats were exposed to periodic administration of 90 min of 100% O_2_ breathing, 5 days/week for 4 weeks (OxCy). Previously, we showed that treatment of MI hearts with bone marrow-derived MSC increased myocardial oxygenation and further that combination treatment of stem cells and OxCy resulted in a higher recovery of cardiac function and cardiomyocyte survival when compared to untreated MI hearts (Khan et al, 2009, 2012). Since p53 has been implicated in cardiomyocyte apoptosis in MI hearts, we determined its role in the oxygenated MI hearts. p53 mRNA and protein levels were determined by RT-PCR, qPCR, Western blotting, *in vivo* ELISA and immunoprecipitation (IP) methods. The results showed that p53 mRNA and protein expression levels were high in both infarct (MI) and oxygenated (MI + OxCy) hearts, whereas control (healthy) hearts did not show p53 gene/protein activation (Fig 1A–E). Since nuclear localization of p53 is critical for its transcriptional activity, we analysed subcellular localization of p53 in the heart tissues by fractionation followed by IP as described (Gogna et al, 2012a). The purity of the fractions was verified using immunoprecipitation with PARP and tubulin antibodies; PARP antibody stains only the nuclear fraction and not the cytoplasmic fraction, while tubulin antibody stains only the cytoplasmic fraction (Supporting Information Fig S1). The IP data of heart tissue showed nuclear migration of p53 in both MI and oxygenated hearts (Fig 1F). The stability of p53 protein was determined in the infarct hearts by probing the formation of p53-Mdm2 complex. Co-IP using both anti-p53 and anti-Mdm2 antibodies showed absence of p53-Mdm2 interaction in both MI and oxygenated hearts (Fig 1G), suggesting active p53 status in both cases. The transcriptional activity of p53 was determined by analysing p53-p300 transcriptional complex. Co-IP using both anti-p53 and anti-p300 antibodies showed that p53 forms a protein-protein complex with its co-activator p300 in the infarct and oxygenated hearts (Fig 1H). Overall, the results indicated that p53 protein was active, stable and transcriptionally potent in both untreated MI and oxygenated MI hearts suggesting a possible mechanism of p53 participating in antagonistic pathways of cardiomyocyte death or survival.

### p53 transcriptionally regulates NOS3 promoter by binding at NOS3-response element

**Figure 2:**
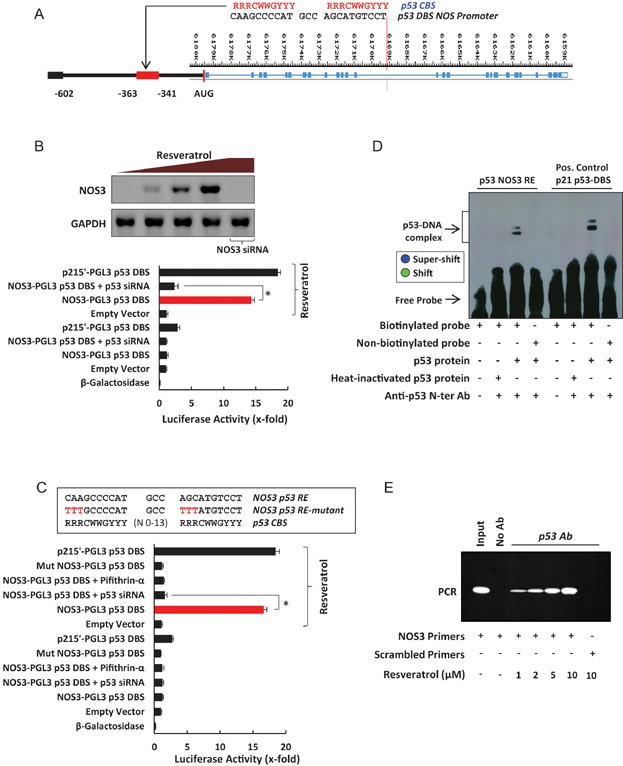
NOS3 is direct transcriptional target of p53 A putative p53 binding site was identified in the NOS3 promoter using Genomatix, MatInspector module. NOS3-p53RE lies between −363 and −341 bp on the 602-bp NOS3 promoter.pNOS3p-luc1 (NOS3 602-bp promoter luciferase construct) was transfected in L6 cells and the effect of p53 on luciferase activity was measured. Resveratrol was used to activate p53 protein (Western blot). p53 gene-activation induces a 14-fold increase in pNOS3p-luc1 luciferase activity (red bar). p53 gene-silencing using p53 siRNA reverses this effect. Data represent mean ± SD of six independent measurements. *p = 2.0E−12. The results show that p53 transcriptionally regulates NOS3 promoter.pNOS3p-luc2, the NOS3-p53RE (−363 to −341) cloned in luciferase vector was transfected in L6 cells in presence of resveratrol to activate p53 protein. Results show that p53 induces a 16-fold increase in the NOS3-p53RE luciferase activity (red bar). p53 gene-silencing and p53 transcriptional inhibition using p53 siRNA and pifithrin-α abolishes the increase in luciferase activity. Data represent mean ± SD of six independent measurements. *p = 8.0E−12. The results show that p53 regulates NOS3 promoter via NOS3-p53RE. The mmpNOS3p-luc2 construct (mmp-mutant minimal promoter) with mutated sequence of NOS3-p53RE was transfected in resveratrol-treated L6 cells. No increase in the luciferase activity is observed, showing the specificity of NOS3-p53RE.Interaction of p53 with NOS3-p53RE is confirmed using EMSA, results show presence of shift and super-shift, p215'RE is used as a positive control.Chromatin immunoprecipitation (ChIP) was conducted in resveratrol-treated L6 cells to confirm *in vivo* binding of p53 to NOS3-p53RE. p53 shows an increase in NOS3-p53RE binding with increasing dose of resveratrol. Scrambled primers for PCR were used as negative control.

Since the p53 levels in the MI and oxygenated MI hearts showed a positive correlation with NOS3 expression profiling (Khan et al, 2009, 2012), it was of interest to define the role of p53 in the regulation of NOS3 and NOS3-mediated cardioprotection. From matrix matches determined by MatInspector (Genomatix), we identified a NOS3 promoter region in the rat NOS3 gene (chromosome 4: 6158847–6179441; reverse strand) as a 602-bp DNA sequence, upstream of +1 transcription start site, in the region 6174620–6175221. We further identified a putative p53 DNA-binding site in the NOS3 promoter region (Fig 2A) using bioinformatics analysis of MatInspector genomatix database (matrix sim; score >0.9). The finding suggested that p53 might be a potential NOS3 regulator. To establish this, we cloned the 602-bp putative NOS3 promoter carrying the p53 response element (p53RE) into a pGL3 basic vector to generate pNOS3p-luc1 (Supporting Information Fig S2). The pNOS3p-luc1 was transfected in L6 cells and treated with resveratrol, a known activator of p53 and NOS3 (Kim et al, 2009). The results showed that p53 increased the activity of NOS3 promoter by 12-fold in the resveratrol-treated cells, when compared to untreated cells (Fig 2B). p53 gene-silencing using p53 siRNA reduced NOS3 promoter activity to 2-fold, when compared to untreated cells. Similarly, p53 also increased NOS3 promoter activity in H4TG hepatoma cells (Supporting Information Fig S3). We further observed that p53-silencing in resveratrol-treated L6 cells led to a decrease in NOS3 mRNA and protein expressions (Supporting Information Fig S4). The results established that the resveratrol-mediated NOS3 activation was due to the action of p53 on NOS3 promoter.

To further confirm the involvement of p53RE in NOS3 promoter, we cloned the −341 to −363 region of NOS3 promoter carrying the p53RE into a pGL3 vector to generate the minimal pNOS3p-luc2 (Supporting Information Fig S5). This NOS3 minimal promoter was induced upon p53 transfection and resveratrol treatment (Fig 2C). Pifithrin-α, an inhibitor of p53 transcriptional activity, abolished NOS3 promoter activity suggesting p53-mediated transcriptional regulation of NOS3 gene. The p53RE sequence was mutated and cloned in PGL3 vector to generate a mutant minimal mmpNOS3p-luc2 (Supporting Information Fig S5). Transfection of mmpNOS3p-luc2 showed no increase in the NOS3 promoter activity (Fig 2C). Analysis of p53-binding to NOS3 promoter by EMSA using p53 protein extracted from untreated and resveratrol-treated L6 cells and NOS3-p53RE sequence (23-bp) confirmed direct binding between NOS3-p53RE and p53 (Fig 2D). To further define the role of NOS3-p53RE in p53-mediated NOS3 induction, we performed chromatin immunoprecipitation (ChIP) assays in L6 cells treated with increasing doses of resveratrol. Consistent with luciferase and EMSA results, we detected one specific PCR product derived from NOS3-p53RE (Fig 2E). These results established that NOS3-p53RE was responsible for the p53-mediated induction of NOS3 promoter activity and that p53 transcriptionally induced NOS3 through promoter binding. Since p53 appears to regulate NOS3 expression, we analysed the co-localization of p53 and NOS3 in the control, MI and MI + OxCy hearts. The results (Supporting Information Fig S6) showed co-localization of p53 and NOS3 in the oxygenated MI hearts.

### p53 differentially binds at the BAX and NOS3 gene promoters in the MI and oxygenated MI hearts

**Figure 3:**
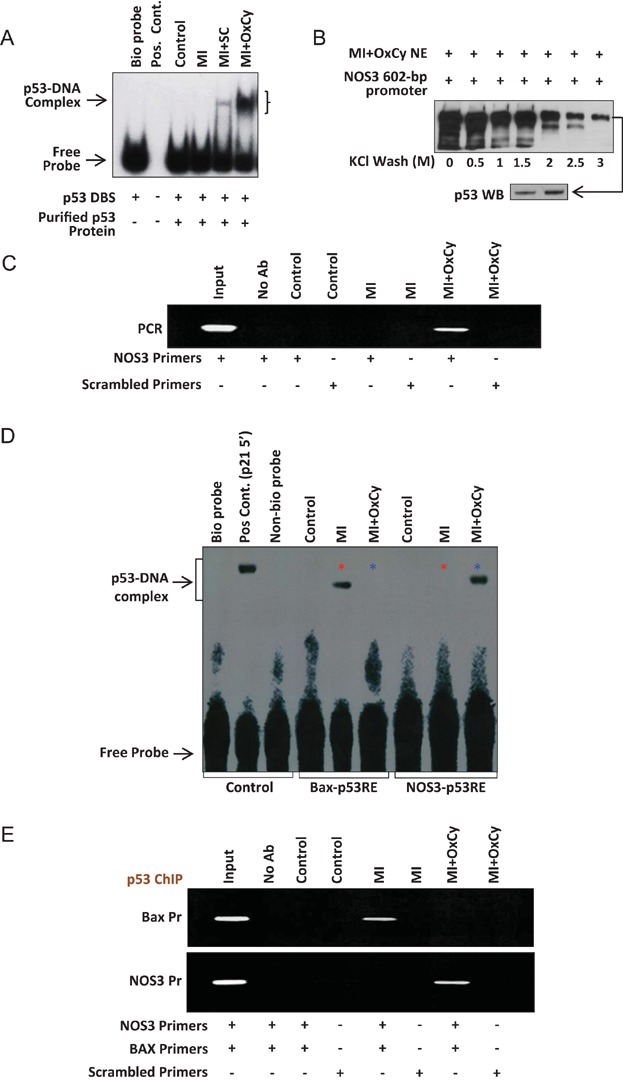
p53 protein from MI and MI + OxCy hearts show differential binding to NOS3-p53RE The binding affinity of p53 protein isolated from control (healthy), MI and MI + OxCy hearts towards NOS3-p53RE was analysed using EMSA. p53 proteins isolated from control and MI hearts show no binding to p53RE in NOS3 promoter, while p53 proteins isolated from MI + OxCy hearts show high affinity towards NOS3-p53RE.Binding affinity of p53 from MI + OxCy was measured using promoter-binding assay. Nuclear extract (NE) of MI + OxCy was incubated with biotin-labelled NOS3-p53RE and the extract was washed using increasing concentration of KCl. Washing of the nuclear extract and biotin-labelled DNA complex with 3M KCl shows a single band in silver staining. The single protein band was eluted from the gel and stained with p53 antibody using Western blotting. The data show that p53 from MI + OxCy hearts have high affinity and specificity for NOS3-p53RE.ChIP assay was performed to confirm differential binding of p53 to NOS3-p53RE in MI and oxygenated hearts. The data show that p53 does not bind to NOS3-p53RE in healthy and infarcted hearts. On the other hand, oxygenation induces p53 binding to the NOS3-p53RE. Scrambled primers were used as negative control and input was used as a positive loading control.Binding of p53 protein isolated from control, MI and MI + OxCy hearts with NOS3-p53RE and BAX-p53RE was analysed using EMSA. p53 isolated from healthy and MI hearts show no binding to NOS3-p53RE whereas p53 isolated from MI + OxCy hearts show very high binding towards NOS3-p53RE. On other hand, p53 isolated from MI hearts show high binding to p53RE in BAX promoter and p53 binding to the BAX-p53RE drops substantially in MI + OxCy treated hearts.ChIP assay in these tissues shows similar results where p53 from MI binds to BAX-p53RE and not to NOS3-p53RE. On the other hand, oxygenation induces shift of p53 binding from BAX to NOS3 promoter.

Since we established that NOS3 is a p53 transcriptional target, we next sought to analyse the role of p53 in NOS3 upregulation in the infarct heart. Previously we showed that oxygenation significantly upregulated the expression of NOS3 in the infarct heart (Khan et al, 2012). p53 was pro-apoptotic in the infarct heart and putatively functioned as a pro-survival factor in the oxygenated heart, suggesting that oxygenation might have altered p53's transcriptional activity. The transcriptional affinity of p53 towards NOS3-p53RE was analysed in the infarct and oxygenated hearts. p53 protein purified from the infarct region of hearts was used to study p53-NOS3-p53RE complex using EMSA. The data showed that p53 from healthy and infarct hearts did not bind to NOS3-p53RE, whereas p53 from oxygenated hearts showed binding to NOS3-p53RE (Fig 3A). p53 from stem cell-transplanted hearts (Khan et al, 2012) were used as control (lane 5). The data suggested that oxygenation transcriptionally modulated p53 to bind to NOS3 promoter. To analyse the specificity and affinity of p53 from oxygenated heart towards NOS3 promoter, NOS3 promoter DNA (602 bp) was incubated with the nuclear extract from oxygenated MI heart. The bound protein was washed using increasing concentrations of potassium chloride to obtain a single band, which was eluted and confirmed to be p53 protein (Fig 3B). The results suggested that oxygenation induced high transcriptional affinity of p53 towards NOS3 promoter. The differential NOS3 promoter binding ability of p53 from healthy, MI and oxygenated MI hearts was further confirmed *in vivo* using ChIP data (Fig 3C), which showed that p53 from healthy and infarct hearts was unable to bind to NOS3-p53RE in the heart. On the other hand, oxygenation induced p53 binding to NOS3-p53RE. The results established that oxygenation modulated p53 transcriptional activity and recruited p53 at NOS3-RE in the infarct heart. Since the MI hearts undergo apoptosis via p53 and its downstream effector protein BAX (Ripa et al, 2006), we analysed if BAX was activated in these hearts. ChIP was conducted to study the binding of p53 at its RE on the BAX promoter as described previously (Kaeser & Iggo, 2002). The results showed significant binding of p53 at the BAX promoter in the MI hearts and this binding was abolished upon oxygenation (Supporting Information Fig S7). BAX mRNA and protein expressions, analysed by RT-PCR and Western blotting, were significantly upregulated in MI hearts and the BAX expression was abolished upon oxygenation of the MI hearts (Supporting Information Figs S8 and S9). This data suggested that if p53 would have to make a choice between the death and survival modes of its activity then oxygenation must revoke the p53-dependent activation of apoptotic genes (*bax*), in addition to p53-dependent NOS3 upregulation. p53 extracted from infarct and oxygenated hearts was used to conduct EMSA to determine the differential binding potential of p53 to NOS3-p53-RE and BAX-p53-RE as described previously (Chou et al, 2011). The results showed that p53 purified from infarct heart, and not from the oxygenated MI heart, was bound to BAX-p53-RE (Fig 3D). On the contrary, p53 from infarct heart showed no binding to NOS3-p53RE, but p53 from oxygenated MI heart showed high affinity for NOS3-p53RE (Fig 3D). This EMSA data showed that, from infarction to oxygenation, p53 transcriptional activity changed from increasing affinity towards NOS3 and decreasing affinity towards BAX gene promoter. The EMSA results were further confirmed *in vivo* using ChIP assay, which showed that p53 from infarct heart was unable to bind to NOS3 promoter and was only bound to BAX promoters. In the oxygenated hearts, p53 showed increased affinity to bind to NOS3 promoter instead of BAX promoter (Fig 3E). Overall, the results seem to suggest that the oxygenation-dependent modulation of p53 transcriptional activity switches p53 from BAX-mediated death effector to NOS3-mediated survival effector.

### Oxygen-induced lack of p53-Lys^118^ acetylation induces p53-NOS3RE interaction and cardiac survival

**Figure 4:**
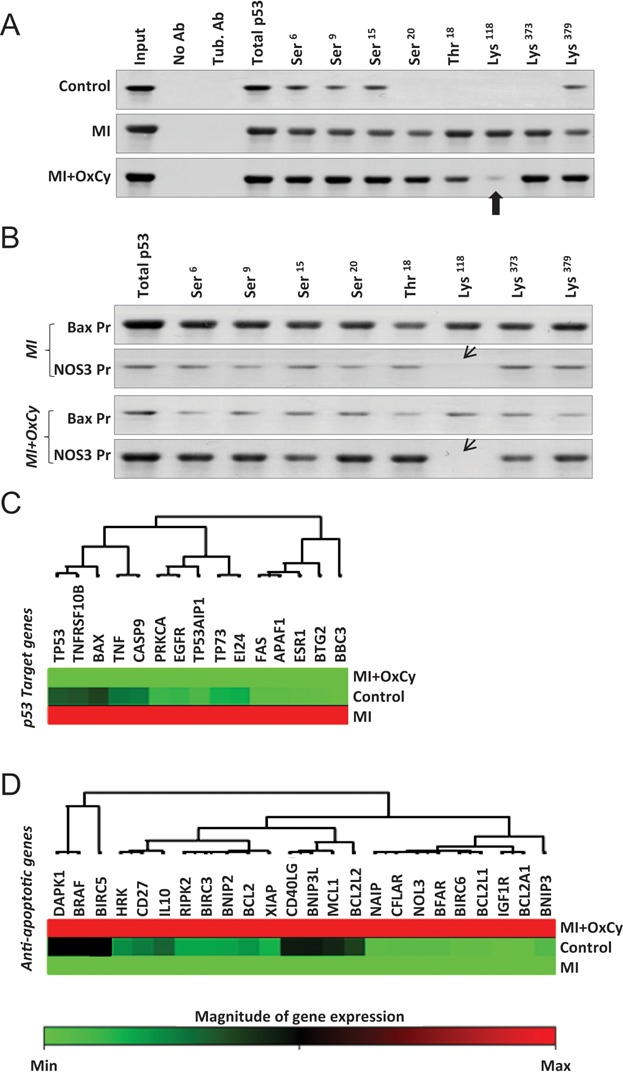
Differential acetylation of p53 at Lys^118^ residue induces differential binding towards BAX and NOS3 promoters in MI and MI + OxCy hearts Status of rat p53 post-translational modifications was analysed by immunoprecipitation of p53 with phosphorylation- and acetylation-specific antibodies. Results show that p53 is phosphorylated and acetylated minimally at Ser^6^, Ser^9^ and Ser^15^, and Lys^379^ in control (healthy) heart. p53 shows very high phosphorylation and acetylation at Ser^6^, Ser^9^, Ser^15^ and Ser^20^, Thr^18^ and Lys^118^, Lys^373^, and Lys^379^ residues in the infarct heart. MI + OxCy heart does not show any post-translational modification of p53 from MI heart other than inducing a decrease in acetylation of Lys^118^ residue.The role of p53-Lys^118^ acetylation in the decision of p53 to bind to BAX-p53RE or NOS3-p53RE status was confirmed by incubating the p53 proteins isolated from MI and MI + OxCy hearts with both BAX-p53RE and NOS-p53RE. The bound fraction of p53 was eluted and probed for p53 posttranslational modifications. Results show that p53 from MI heart is mostly bound to BAX-p53RE and this fraction of p53 is acetylated at Lys^118^ residue. However, a small fraction of p53 from MI heart is also bound to BAX-p53RE but this fraction is not acetylated at Lys^118^ residue. Similarly p53 from MI + OxCy heart is mostly bound to NOS3-p53RE and this fraction is not acetylated at Lys^118^. A small fraction of p53 is found bound to BAX-p53RE and this fraction is acetylated at Lys^118^ residue. The data suggest the importance of Lys^118^ acetylation in deciding the choice of p53 to bind to either BAX or NOS3 promoter, or in other words, to induce death or survival of cardiomyocytes in the infarct hearts.The effect of oxygenation upon the transcriptional activity of p53 in upregulating its downstream apoptotic genes were analysed. The activation of 15 apoptotic genes carrying p53 response element was analysed in the healthy, MI and MI + OxCy hearts. The gene-array data show that the p53 downstream genes involved in apoptosis are activated only in the MI hearts. Upon oxygenation, the p53-mediated activation of these genes is significantly inhibited. These genes show minimal expression in the healthy hearts (control).The expression of 24 anti-apoptotic genes, which might play crucial role in cardiac survival, is observed in the healthy, MI and MI + OxCy hearts. The results show that these genes are minimally expressed in the Infarct hearts (MI). However, upon oxygenation of the MI hearts there is a significant increase in the expression of these genes.

Since oxygenation was established to modulate p53 transcriptional activity, the mechanism of this effect on p53 DNA-binding ability was determined. Post-translational modifications across the length of p53 help determine p53 transcriptional activity (Xu et al, 2003). p53 phosphorylation/acetylation play crucial role in p53 half-life (Kubbutat et al, 1997; Lu, 2010), nuclear localization (Kruse & Gu, 2009; Liang & Clarke, 2001), protein structure (Chehab et al, 1999; Wieczorek et al, 1996) and transcriptional activity (Brooks & Gu, 2003; Lee et al, 2006). On the basis of our understanding on the role of p53 post-translational modifications in p53 transcriptional activity, we hypothesized that differential p53 post-translational modification patterns might govern differential p53 DNA-binding ability in healthy, infarct and oxygenated hearts. Oxygenation might have altered post-translational modification pattern of p53 which existed in infarct heart. We analysed all serine, threonine and lysine motifs of rat p53 that are known to be phosphorylated and acetylated. Healthy, infarct and oxygenated hearts showed qualitative as well as quantitative differential modifications at Ser^6^, Ser^9^, Ser^15^, Ser^20^, Thr^18^, Lys^118^, Lys^373^ and Lys^379^ residues of p53 (Fig 4A). Control (healthy) hearts showed minimal modifications of p53 at Ser^6^, Ser^9^, Ser^15^ and Lys^379^ residues. The results of the IP analysis were repeated using a quantitative *in vivo* ELISA technique as described previously (Gogna et al, 2012b) and shown in Supporting Information Fig S10. p53 in the infarct hearts was phosphorylated and acetylated across all the residues, possibly deciding p53 transcriptional ability toward binding to BAX promoter. Interestingly, oxygenation revoked p53 acetylation at Lys^118^ residue, suggesting that inhibition of Lys^118^ acetylation might act as oxygenation-induced switch, which regulates p53 transcriptional activity and thus p53's ability to activate either pro-death BAX promoter or the pro-survival NOS3 promoter. To confirm the role of Lys^118^ acetylation in p53's decision to bind to BAX or NOS3 promoter, we incubated p53 extracted from infarct and oxygenated hearts with biotin-labelled NOS3-RE and BAX-RE. The bound protein was eluted and analysed for the post-translational modifications existing on the p53 bound to BAX-RE and NOS3-RE using IPP. The results showed a small fraction of p53 purified from infarct heart was bound to p53-NOS3-RE and that fraction of p53 was not acetylated at Lys^118^ residue (Fig 4B). This data confirmed that infarction induced apoptosis and BAX activation through Lys^118^ acetylation, and if Lys^118^ acetylation was removed then the same p53 has affinity for NOS3 promoter. Thus, conversion of p53 transcriptional activity and p53 effector response from death to survival in rat heart was dependent on Lys^118^ acetylation. Similarly, the p53 purified from oxygenated MI heart was incubated with BAX-p53-RE and NOS3-p53-RE and bound p53 was analysed for the post-translational modifications. The data further established that a small fraction of cellular p53 was bound to the BAX promoter and that fraction was acetylated at Lys^118^ residue. More than 90% of p53 was bound to the NOS3-p53-RE and that fraction was not acetylated at Lys^118^ residue. Next, we used PCR gene-array to determine the expression of 15 genes with p53-RE acting downstream of the transcription factor and involved in apoptosis. The results showed that p53 activated these genes only in the MI hearts and these genes were abolished upon oxygenation, suggesting towards an anti-apoptotic/survival pathway (Fig 4C). Next we determined the activation of 24 genes involved in the anti-apoptotic pathway. The gene-array analysis showed that these anti-apoptotic gens were switched off in the MI hearts and they were activated upon oxygenation of these hearts, suggesting towards the existence of a survival pathway in these hearts (Fig 4D).

**Figure 5:**
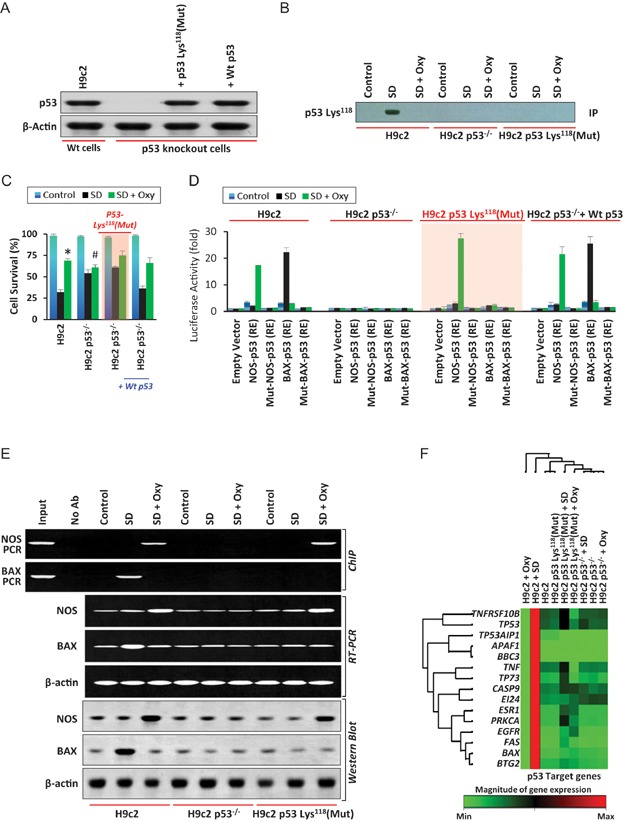
Oxygenation inhibits acetylation of p53-Lys^118^ residue and increases survival of cardiomyocytes p53 knockout and p53^Lys118–Ala118^ mutant H9c2 cells were created as described previously (Gogna et al, 2012c). Western-blot analysis shows the knockout of p53 in H9c2 cells (lane 2), while addition of p53^Lys118–Ala118^ cDNA or p53 Wt cDNA to the H9c2 cells show the expression of p53 (lanes 3,4).The H9c2, H9c2 p53^−/−^ and H9c2 p53-Lys^118^(Mut) cells were cultured under serum-deprived (SD) conditions to mimic MI. p53-Lys^118^ acetylation was analysed in the SD and oxygenated (Oxy) cells by Western blot (WB) and immunoprecipitation (IP) using p53-Lys^118^ antibody.The survival potential of H9c2, H9c2 p53^−/−^, H9c2 p53-Lys^118^(Mut) and H9c2 p53^−/−^ +p53 Wt-cDNA cells was determined in the control, SD and SD + Oxy treatments. Data represent mean ± SD of eight independent measurements. *p = 6.6E−14 *versus* respective SD group; ^#^*p* = 0.0014 *versus* respective SD group. The results show that oxygenation restored the SD-induced cell death.Results of luciferase assay showing the activation of p53-NOS3-RE and p53-BAX-RE in H9c2 cells. Oxygenation of SD cells switches from p53-BAX-RE to p53-NOS3-RE activation. Data represent mean ± SD of eight independent measurements.ChIP assay showing the binding of p53 to its respective NOS3 and BAX RE in H9c2 cells. The data show that p53 binds to BAX-RE in SD cells and shifts its binding to NOS3 upon oxygenation. No binding to either NOS3-RE or BAX-RE is observed in H9c2 p53^−/−^ cells. In H9c2 p53-Lys^118^(Mut) cells, SD does not induce binding of p53 to the BAX-RE but upon oxygenation p53 binds to the NOS3-RE. The data suggest that p53-Lys^118^ acetylation is crucial for binding of p53 to BAX-RE and mutation in this site induces p53 binding to the NOS3-RE. Similar results of NOS3 and BAX mRNA and protein upregulation are observed in RT-PCR and the Western-blot assay.Expression of p53 downstream genes involved in apoptosis in H9c2 cells. The data show that SD results in the activation of these genes, whereas p53^−/−^ and p53-Lys^118^ (Mut) cells do not activate these genes under any condition.

To establish the role of p53-Lys^118^ mutation in the oxygen/p53-initiated survival pathway, we used H9c2 cardiomyocytes and created p53^−/−^ knockdown H9c2 cells and then used the H9c2 p53^−/−^ cells to create H9c2 p53-Lys^118^(Mut) cells by stable transfection of p53 cDNA with Lys^118^-Ala^118^ point mutation(Gogna et al, 2012b). Western-blot analysis confirmed the knockout of p53 in H9c2 p53^−/−^ cells (Fig 5A). The analysis further established that H9c2 p53-Lys^118^(Mut) cells showed expression of p53 protein and H9c2 p53^−/−^ cells transiently transfected with p53 Wt cDNA also expressed the p53 protein (Fig 5A). These H9c2 cells were cultured under serum-deprived (SD) conditions for 72 h (Zheng et al, 2010) to mimic the apoptotic conditions in the MI hearts. Following serum deprivation, these cells were oxygenated at 60% O_2_ for 24 h to mimic MI + OxCy condition. The status of p53-Lys^118^ acetylation was analysed in these modified cardiomyocytes using IP. The results showed that SD in H9c2 cells induced acetylation of p53-Lys^118^ residue and oxygenation (SD + Oxy) abolished this acetylation (Fig 5B). Acetylation of p53-Lys^118^ residue was not observed in H9c2 p53^−/−^ and H9c2 p53-Lys^118^(Mut) cells. The effect of SD and SD + Oxy treatments on the survival of these modified cardiomyocytes was studied using Annexin-V staining. The results (Fig 5C) showed that upon SD treatment H9c2 p53^−/−^ and H9c2 p53-Lys^118^(Mut) cells showed higher survival than H9c2 (Wt) cells, suggesting that lack of p53 acetylation at Lys^118^ residue induced survival in the SD-treated cells. We next determined the transcriptional activity of p53 at the BAX-RE and NOS3-RE in these cells. SD treatment activated BAX-p53-RE and SD + Oxy treatment activated NOS3-p53-RE (Fig 5D). In H9c2 p53^−/−^ cells, no activation of either BAX-p53-RE or NOS3-p53-RE was observed. In H9c2 p53-Lys^118^(Mut) cells, only the NOS3-p53-RE was activated upon SD + Oxy treatment, but the BAX-p53-RE was not activated suggesting the importance of p53-Lys^118^ acetylation in the activation of apoptotic BAX. H9c2 p53^−/−^ cells transiently transfected with WT-p53 cDNA were used as control. To relate the gene activation with the direct binding of p53 to its BAX-RE or the NOS3-RE, ChIP assay was conducted in H9c2 cells. The results showed that p53 was bound to BAX-RE in SD-treated cells and this binding was abolished upon oxygenation (Fig 5E). Similarly, p53 was bound to NOS3-RE in SD + Oxy treated cells. No binding to either BAX-RE or the NOS3-RE was observed in the H9c2 p53^−/−^ cells. In H9c2 p53-Lys^118^(Mut) cells, p53 was bound to only NOS3-RE and its binding with BAX-RE was abolished even in the SD-treated cells. The mRNA and protein expression of BAX and NO3 in these cardiomyocytes also showed similar pattern of gene expression, where in the H9c2 p53-Lys^118^(Mut) cells only NOS3 gene was activated and BAX was not upregulated even upon SD treatment. Further, the activation of 15 p53 target apoptotic genes was analysed in these modified cardiomyocytes and results showed that only H9c2 cells upon SD treatment activated the apoptotic genes (Fig 5F). Neither the H9c2 p53^−/−^ cells or the H9c2 p53-Lys^118^(Mut) cells showed any activation of the apoptotic genes upon SD or SD + Oxy treatment.

**Figure 6:**
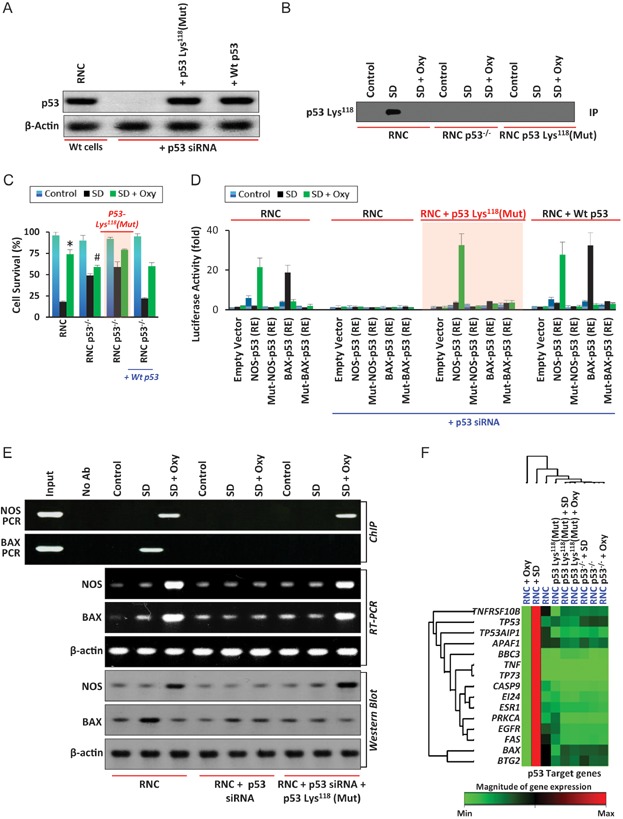
Oxygenation inhibits acetylation of p53-Lys^118^ residue and increases survival of rat neonatal cardiomyocyte (RNC) cells Western-blot results show the abolition of p53 expression in RNC cells (lane 2), while addition of p53^Lys118-Ala118^ cDNA and p53 Wt cDNA to RNC p53^−/−^ cells show the expression of p53 (lanes 3 and 4). Control cells show expression of p53 (lane 1).Immunoprecipitation results show acetylation of p53 at ^Lys118^ residue in RNC cells under SD conditions (lane 2). However, this expression is abolished upon oxygenation (lane 3). Acetylation at p53 ^Lys118^ is absent in control, RNC p53^−/−^ and RNC p53-Lys^118^(Mut) cells under both SD and SD + Oxy conditions (lanes 4–9).Cell survival analysis of control, SD and SD + Oxy-treated RNC, RNC + p53 siRNA, RNC + p53 siRNA + p53-Lys^118^(Mut) cDNA and RNC + p53 siRNA + Wt p53 cDNA cells show an increase in the SD-treated RNC + p53 siRNA and RNC + p53 siRNA + p53-Lys^118^(Mut) cDNA cells compared to RNC cells, exhibiting the crucial role of deacetylation of p53 Lys^118^ in cell survival. Data represent mean ± SD of eight independent measurements. *p = 2.6E−14 *versus* respective SD group; ^#^*p* = 9.3E−08 *versus* respective SD group.Luciferase assay shows activation of BAX-p53-RE and NOS3-p53-RE in SD and SD + Oxy-treated RNC cells. Neither BAX nor NOS3 is activated in RNC + p53 siRNA cells. Oxygenation caused a switch of BAX activation to NOS3 activation in RNC p53-Lys^118^(Mut) cells. Data represent mean ± SD of eight independent measurements.ChIP assay shows the binding of p53 to its respective NOS3 and BAX RE in RNC cells (lanes 4 and 5). However, no binding of p53 to BAX-RE or NOS3-RE is observed in RNC p53^−/−^ cells (lanes 7 and 8). However, p53 binds to NOS3-RE in SD + Oxy-treated RNC p53-Lys^118^(Mut) cells (lane 11). These results suggest the importance of p53 ^Lys118^ acetylation in activation of either BAX-RE or NOS3-RE and regulation of apoptotic or survival pathway respectively. Similar results were observed at mRNA and protein level using RT-PCR and Western-blot techniques in RNC, RNC p53^−/−^ and RNC p53-Lys^118^(Mut) cells.Expression of p53 downstream genes in RNC, RNC p53^−/−^ and RNC p53-Lys^118^(Mut) cells. SD-treated RNC cells show activation of apoptotic genes whereas RNC p53^−/−^ and RNC p53-Lys^118^(Mut) cells show no involvement in apoptotic gene activation.

Similar set of experiments were carried out using rat neonatal cardiomyocytes (RNC). p53 siRNA was used to abolish p53 expression in RNC cells and further these cells were used to generate RNC p53-Lys^118^(Mut) cells upon stable transfection with p53 cDNA carrying Lys^118^-Ala^118^ point mutation (Gogna et al, 2012b). Western-blot analysis confirmed successful abolition of p53 expression in RNCs (Fig 6A; lane 2). However, both RNC p53-Lys^118^(Mut) cells and RNC p53^−/−^ cells transiently transfected with p53 Wt cDNA, showed expression of p53 protein (Fig 6A; lanes 3 and 4). The cells were cultured under SD conditions for 72 h (Zheng et al, 2010) in order to mimic the apoptotic conditions as in MI hearts and further oxygenated at 60% O_2_ for 24 h to mimic MI + OxCy conditions. Immunoprecipitation using p53-Lys^118^ acetylation antibody showed acetylation at p53 Lys^118^ residue in RNC cells subjected SD while the expression was abrogated upon oxygenation (SD + Oxy; Fig 6B; lanes 2 and 3). Acetylation of p53-Lys^118^ residue was absent in RNC p53^−/−^ and RNC p53-Lys^118^(Mut) cells (Fig 6B; lanes 4–9). Effect of SD and SD + Oxy treatments on cell survival was determined in RNC, RNC p53^−/−^ and RNC p53-Lys^118^(Mut) cells using Annexin V staining. SD-treated RNC p53^−/−^ and RNC p53-Lys^118^(Mut) cells showed higher survival compared to RNC (Wt) cells (Fig 6C), suggesting the importance of deacetylation of p53 at Lys^118^ residue in cell survival. The transcriptional activity of p53 at the BAX-RE and NOS3-RE was determined in the RNC, RNC p53^−/−^ and RNC p53-Lys^118^(Mut) cells using luciferase activity. The results showed activation of BAX-p53-RE and NOS3-p53-RE upon SD and SD + Oxy treatment, respectively, in RNC cells (Fig 6D). Neither BAX-p53-RE nor NOS3-p53-RE was activated in RNC p53^−/−^ cells. In RNC p53-Lys^118^(Mut) cells, SD treatment showed no activation of BAX-p53-RE upon SD treatment but showed activation of NOS3-p53-RE upon SD + Oxy treatment, suggesting the importance of p53-Lys^118^ acetylation in the activation of apoptotic BAX. RNC p53^−/−^ cells transiently transfected with WT-p53 cDNA were used as control. In order to correlate the gene activation with the direct binding of p53 to its BAX-RE or the NOS3-RE, ChIP assay was conducted in RNC, RNC p53^−/−^ and RNC p53-Lys^118^(Mut) cells. The assay showed binding of p53 to BAX-RE and NOS3-RE in RNC cells subjected to SD and SD + Oxy treatments, respectively (Fig 6E). On the other hand, p53 showed no binding to either BAX-RE or the NOS3-RE in RNC p53^−/−^ cells. RNC p53-Lys^118^(Mut) cells showed p53 bound only to NOS3-RE upon SD + Oxy treatment. Western-blot and RT-PCR analyses showed upregulation of BAX and NOS genes upon SD and SD + Oxy treatment, respectively, in RNC cells. In RNC p53-Lys^118^(Mut) cells only NOS3 gene was upregulated upon SD + Oxy treatment while BAX expression was not upregulated even upon SD treatment. In addition, SA Biosciences Apoptosis kit was used to study the activation of 15 p53 target apoptotic genes in the RNC, RNC p53^−/−^ and RNC p53-Lys^118^(Mut) cells. The data showed that RNC cells alone activated the apoptotic genes upon SD treatment (Fig 6F). However, neither the RNC p53^−/−^ cells nor RNC p53-Lys^118^(Mut) cells showed any activation of the apoptotic genes upon SD or SD + Oxy treatment.

### Oxygen inhibits p53-Lys^118^ acetylation by negatively regulating TIP60 acetylase in infarct hearts

**Figure 7:**
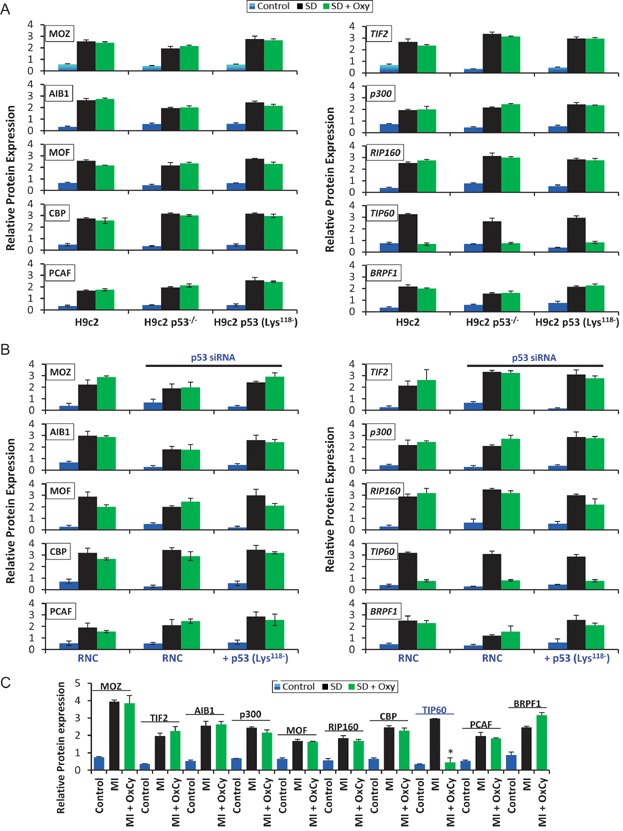
Oxygenation inhibits TIP60 expression in MI heart Expression of p53-interating acetylases in the control, serum-deprived (SD), and oxygenated (SD + Oxy) H9c2, H9c2 p53^−/−^, H9c2 p53-Lys^118^(Mut) cells, as determined by *in vivo* ELISA.Expression of p53-interating acetylases in the control, serum-deprived (SD), and oxygenated (SD + Oxy) RNC, RNC p53^-/-^, RNC p53-Lys^118^(Mut) cells. The results show that the expression of TIP60, a p53-interacting acetylase, is inhibited upon oxygenation in these cells.Inhibition of TIP60 expression is also observed in the MI + OxCy heart tissues. Data represent mean ± SD of eight independent measurements in all groups. *p = 6.2E−12 *versus* respective MI group.

**Figure 8:**
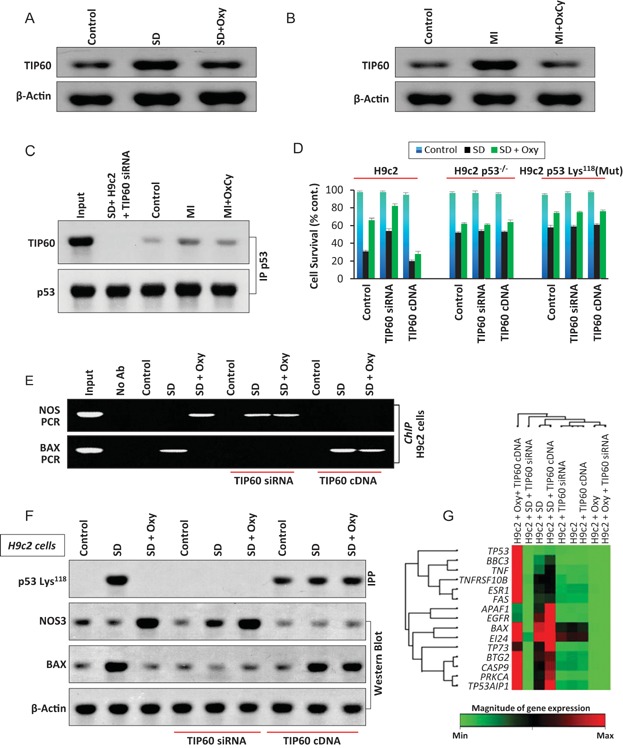
TIP60 regulates p53-mediated activation of BAX or NOS3 gene in H9c2, H9c2 p53^−/−^ and H9c2 p53-Lys^118^ (Mut) cells TIP60 protein expression in H9c2 cells under control, SD, and SD + Oxy conditions. SD induces TIP60 expression, which is inhibited by oxygenation.TIP60 expression in MI hearts is inhibited by oxygenation.Co-immunoprecipitation of p53 with TIP60 shows that TIP60 binds to p53 only in MI hearts and this interaction is abolished upon oxygenation. Input and SD-treated H9c2 cells with TIP60 gene-silencing were used as controls.Effect of TIP60 on the survival of SD H9c2 cells. Silencing of TIP60 using TIP60 siRNA in H9c2, H9c2 p53^−/−^ and H9c2 p53-Lys^118^(Mut) cells increases the survival of SD cells. Addition of TIP60 using cDNA results decreases the survival of SD cells. Data represent mean ± SD of four independent measurements.Effect of TIP60 on the binding of p53 to BAX-RE and NOS3-RE was analysed using ChIP in H9c2 cells. Results show that TIP60 gene-silencing results in binding of p53 to NOS3-RE in both SD and SD + Oxy cells. Similarly, exogenous addition of TIP60 cDNA results in binding of p53 to BAX-RE in both SD and SD + Oxy cells.Effect of TIP60 siRNA and TIP60 cDNA addition on p53-Lys^118^ acetylation, NOS3 and BAX protein expression was determined using Western blot. Data show that TIP60 siRNA abolishes p53-Lys^118^ acetylation, increases the expression of NOS3 protein and decreases BAX expression in H9c2 cells. Similarly, TIP60 cDNA increases p53-Lys^118^ acetylation, decreases the expression of NOS3 protein and increased BAX expression in H9c2 cells.Expression of p53 downstream genes was determined in the SD and SD + Oxy group of H9c2 cells in presence and absence of TIP60 siRNA and TIP60 cDNA. Data show that TIP60 cDNA increases the expression of these genes and TIP60 siRNA decreases their expression in both SD and SD + Oxy groups.

We studied the mechanism through which oxygenation of the cardiomyocytes and the infarct heart results in the suppression of p53-Lys^118^ acetylation. We had previously established a set of 10 p53 acetylases, which are involved in the acetylation of p53 at its various known residues (Gogna et al, 2012c). *In vivo* ELISA was conducted in H9c2, H9c2 p53^−/−^ and H9c2 p53-Lys^118^(Mut) cells to check the expression-status of MOZ, TIF2, AIB1, p300, MOF, RIP160, CBP, TIP60, PCAF and BRPF1 acetylases. The results showed that these acetylases were activated upon SD treatment (Fig 7A). Oxygenation of these SD-treated cells did not result in their inhibition. However, TIP60 showed significant regression upon oxygenation. Same set of experiment was repeated with RNC, RNC p53^−/−^ and RNC p53-Lys^118^(Mut) cells and consistent results were obtained (Fig 7B). Expression of these acetylases was also determined in Control, MI and MI + OxCy heart tissues. *In vivo* ELISA results showed that, amongst all other acetylases, only TIP60 expression was significantly reduced in the MI + Oxy heart (Fig 7C). Western-blot analysis of TIP60 was conducted to establish its role in the oxygen-mediated un-acetylation of p53-Lys^118^ residue. The results showed that SD treatment in the H9c2 cells increased the expression of TIP60 protein and oxygenation decreased its expression (Fig 8A). Similarly, in cardiac tissue the MI hearts showed a high expression of TIP60 and the TIP60 expression was decreased in the MI + OxCy hearts (Fig 8B). The binding between p53 and its acetylase TIP60 was analysed using co-immunoprecipitation. The results showed that TIP60 interacted with p53 in the MI hearts and this interaction was abolished upon oxygenation of these hearts (Fig 8C). The role of TIP60 in the survival of SD and SD + Oxy-treated cardiomyocytes was studied via Annexin-V staining. The results showed that silencing TIP60 gene using TIP60 siRNA resulted in an increase in the survival of SD-treated H9c2 cells (Fig 8D). Exogenous addition of TIP60 cDNA resulted in a significant increase in these cardiomyocyte death and even SD + Oxy treatment could not prevent cells from apoptosis. The addition of TIP60 siRNA or TIP60 cDNA in H9c2 p53^−/−^ and H9c2 p53-Lys^118^(Mut) cells had no effect in the regulation of apoptosis in these cardiomyocytes. ChIP analysis on the BAX-RE and the NOS3-RE showed that TIP60 siRNA induced binding of p53 to NOS3-RE even in the SD-treated cells (Fig 8E; lanes 7 and 8). In H9c2 cells with exogenous addition of TIP60 cDNA, p53 was observed to bind to BAX-RE in both SD and SD + Oxy-treated cells (Fig 8E; lanes 10 and 11). Western-blot analysis of p53-Lys^118^ acetylation, NOS3 and BAX showed that TIP60 siRNA abolished p53-Lys^118^ acetylation, increased the synthesis of NOS3 protein, and inhibited BAX synthesis in both SD and SD + Oxy-treated H9c2 cells (Fig 8F; lanes 4–6). Similarly, in H9c2 cells, where TIP60 cDNA was ectopically expressed, the p53-Lys^118^ acetylation was observed along with lack of NOS3 synthesis and abundant BAX synthesis (Fig 8F; lanes 7–9). PCR gene-array analysis showed that the addition of TIP60 cDNA resulted in the activation of these p53-regulated apoptotic genes in both SD and SD + Oxy treatments. In H9c2 cells expressing the TIP60 siRNA, the expression of these apoptotic genes was inhibited even in the SD treatment.

**Figure 9:**
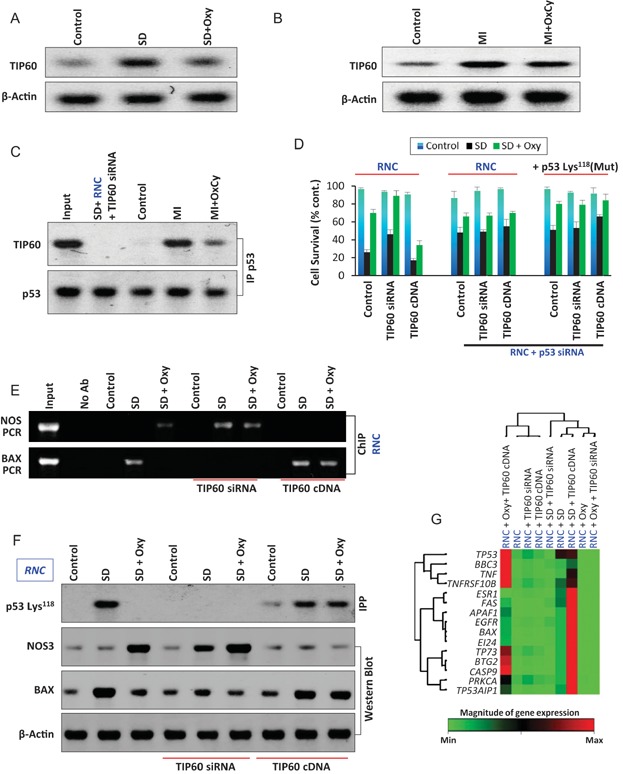
TIP60 regulates p53-mediated activation of BAX or NOS3 gene in RNC, RNC p53^−/−^ and RNC p53-Lys^118^(Mut) cells Western-blot analysis was conducted to study the expression of TIP60 in control, SD and SD + Oxy-treated cells. SD induces TIP60 expression (lane 2); however, this expression is significantly inhibited by oxygenation (lane 3).Western blots of TIP60 expression in MI and MI + Oxy-treated cardiac tissue. Results show increased TIP60 expression in MI tissue (lane 2); however, this expression is significantly reduced in MI + Oxy-treated cardiac tissue (lane 3).Co-immunoprecipitation study was conducted to study p53 and TIP60 interaction in MI and MI + Oxy-treated RNC cells. Results show p53 interaction with TIP60 in SD-treated cells (lane 4); however, this oxygenation significantly inhibits this interaction (lane 5) in RNC cells. Input and TIP60 siRNA were used as controls for the study.The role of TIP60 in cell survival was studied in RNC cells using flow cytometry. Transfection with TIP60 siRNA results in increase in cell-survival fraction in RNC cells while TIP60 cDNA results in a decrease in cell-survival fraction. Conversely, neither TIP60 siRNA nor TIP60 cDNA has any effect on cell survival in RNC p53^−/−^ and RNC p53-Lys^118^(Mut) cells. Data represent mean ± SD of four independent measurements.CHIP assay was conducted to study the role of TIP60 in p53 binding to BAX-RE or NOS3-RE in SD and SD + Oxy-treated RNC cells. Results show that transfection with TIP60 siRNA results in p53 binding to NOS-RE in both SD and SD + Oxy-treated cells. However, exogenous addition of TIP60 cDNA result in p53 binding to BAX-RE in both SDF and SD + Oxy-treated cells.Effect of TIP60 siRNA and TIP60 cDNA addition on p53-Lys^118^ acetylation, NOS3 and BAX protein expression was studied in SD and SD + Oxy-treated RNC cells. Transfection with TIP60 siRNA led to abolition of p53-Lys^118^ acetylation, increased NOS3 expression and decreased BAX expression in RNC cells. Contrarily, transfection with TIP60 cDNA led to increased p53-Lys^118^ acetylation, decreased NOS3 expression and increased BAX expression in RNC cells.Gene array was studied to study the role of TIP60 in activation of p53 downstream genes in SD and SD + Oxy treated cells. Transfection with TIP60 cDNA results in the activations of apoptotic genes in SD and SD + Oxy-treated cells when compared to TIP60 siRNA-transfected SD and SD + Oxy-treated groups.

Mechanism of oxygen-mediated deacetylation of TIP60 and activation of NOS3 based cardioprotective survival pathway was further confirmed in RNC, RNC p53^−/−^ and RNC p53-Lys^118^(Mut) cells. Western blot showed higher level of TIP60 expression in RNCs subjected to SD when compared to SD + Oxy treatment (Fig 9A). Similar results were obtained in MI and MI + OxCy cardiac tissues (Fig 9B). Co-immunoprecipitation analysis showed increased TIP60 interaction with p53 in the MI tissue contrary to oxygenated MI tissue (Fig 9C). The effect of TIP60 on the survival of SD and SD + Oxy-treated RNC, RNC p53^−/−^ and RNC p53-Lys^118^(Mut) cells was determined using Annexin-V staining. TIP60 siRNA transfection resulted in an increase in the survival RNC cells (Fig 9D). Conversely, TIP60 cDNA resulted in a decrease in cell survival. However, neither TIP60 siRNA or TIP60 cDNA transfection affected the cell survival in RNC p53^−/−^ and RNC p53-Lys^118^(Mut) cells. ChIP assay showed that p53 was bound to NOS-RE in SD-treated RNC cells transfected with TIP60 siRNA (Fig 9E; lanes 7 and 8). However, p53 was bound to BAX-RE in both SD and SD + Oxy-treated RNC cells exogeneously transfected with TIP60 cDNA, (Fig 9E; lanes 10 and 11). Western-blot studies were conducted to study the expression of p53-Lys^118^ acetylation, NOS3 and BAX proteins in SD and SD + Oxy-treated RNC cells upon TIP60 siRNA and TIP60 cDNA transfection. Ectopical expression of TIP60 cDNA resulted in increased expression of acetylated p53 at Lys^118^ residue, decreased NOS3 and increased BAX expression upon SD and SD + Oxy treatment (Fig 9F; lanes 5,6,8,9). Consistent with the above results, an increase in NOS3 expression was observed in SD-treated RNC cells transfected with TIP60 siRNA (Fig 9F; lanes 5 and 6). Gene-array analysis was used to study the effect of TIP60 in the regulation of p53 downstream apoptotic genes in both SD and SD + Oxy-treated RNC cells. SD and SD + Oxy-treated RNC cells showed activation of apoptotic genes upon transfection with TIP60 cDNA, however, this effect was reversed in SD and SD + Oxy-treated cells upon transfection with TIP60 siRNA (Fig 9G). This data suggested that oxygen inhibits TIP60 expression in the infarct myocardium, which result in the lack of acetylation of p53 protein at the Lys^118^ residue, which is responsible for the activation of NOS3-based cardioprotective/survival pathway.

## DISCUSSION

The present study, for the first time, establishes a new prosurvival role of p53 for cardioprotection. p53 inhibits apoptosis and protects infarct myocardium under conditions of enhanced oxygenation in the MI heart. In oxygenated infarct heart, p53 is post-translationally modified to support the transcription of NOS3 gene for cardioprotection. Post-conditioning of infarct heart with oxygen suppresses p53's role as an apoptotic effector and switches to promoter of cell survival. The switching of p53's action is evident from a reversal of its affinity for BAX-p53-RE to NOS3-p53-RE, through a molecular switch, which involves regulation of p53 acetylation at Lys^118^ residue in the infarct heart. Our results further suggest that oxygenation of the infarct heart inhibits the expression of TIP60 acetylase and abolishes its interaction with p53 and Lys^118^ acetylation. Lack of acetylation at p53-Lys^118^ residue results in the activation of NOS3 promoter, while suppressing the activation of apoptotic genes including BAX. In MI hearts, where TIP60 is overexpressed, p53 is acetylated at the Lys^118^ residue and binds to the BAX promoter and induces upregulation of BAX protein. Both the p53-knockout cardiomyocytes and cardiomyocytes carrying the p53-Lys^118^ (Mut) protein showed inhibition of TIP60 and suppression of the p53-Lys^118^ acetylation upon oxygenation.

It is well-established that p53 is activated in cancer cells upon receiving cellular/genotoxic stress and meticulously orchestrates to choose its downstream effector responses such as cell-cycle arrest, senescence, apoptosis and DNA repair. For evoking these effector responses, p53 carefully and differentially selects its target genes, which determine the eventual result of p53 activation (Gogna et al, 2012a; Ryan, 2012). In the present study, we have presented evidence that the ability of p53 to differentially choose its target sites in the chromatin not only occurs in cancer cells but is also a possible phenomenon in the cardiac system. In this manuscript, we are proposing a novel downstream effector response of p53 that leads to cell-survival of cardiomyocytes in the infarct heart. We have presented evidence that p53 chooses between the apoptotic and survival pathways depending on the status of its Lys^118^ acetylation.

In human, acetylation of p53-Lys^120^ residue, which lies within the p53 DNA-binding domain, is catalysed by the acetyl-transferase TIP60/KAT5 (Tang et al, 2006). Acetylation of this site is indispensable for p53-dependent apoptosis but p53-Lys^120^ mutants are able to induce p53-mediated transcription and cell-cycle arrest (Sykes et al, 2006; Tang et al, 2006). The Lys^120^-acetylated p53 specifically accumulates at proapoptotic target genes such as BAX and PUMA, whereas a non-acetylated mutant is defective for transcription of these proapoptotic targets. Furthermore, point mutations at Lys^120^ occur in human cancer, including one that converts lysine to arginine. These mutants result in the lack of p53 apoptosis response (Petitjean et al, 2007). A variety of de-acetylases or HDACs (histone de-acetylases) or KDACs (lysine de-acetylases) are known to induce de-acetylation of p53 at a variety of lysine residues at the p53 C-terminus (Yang & Seto, 2008). Lysine residues within the C terminus of p53 can be de-acetylated by either HDAC1 or SIRT1, which affects p53 stability (Barlev et al, 2001; Ito et al, 2002), cofactor recruitment (Gu & Roeder, 1997) and DNA binding (Khan et al, 2008). However, the regulation of the acetylation and de-acetylation pathway of the p53 core domain Lys^120^ residue is not known (Sykes et al, 2006; Tang et al, 2006). It is interesting to note that the single modification at the Lys^120^ residue determines the apoptotic or survival fate of cancer cells.

**Figure 10:**
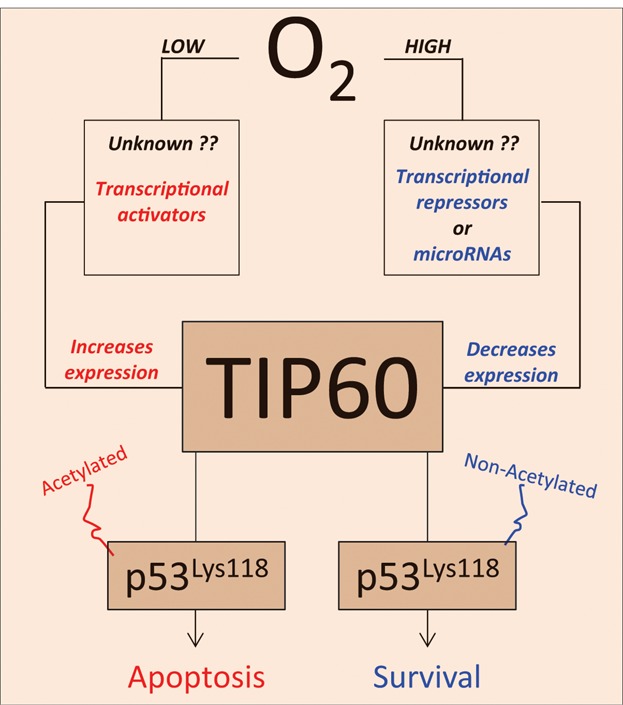
Hypothetical model illustrating how oxygen might regulate p53 transcriptional activity via TIP60 gene regulation.

In the infarct myocardium p53 exists as an apoptotic transcription factor, activates BAX and is heavily phosphorylated and acetylated at its known residues. Interestingly, upon oxygenation of the infarct heart, p53 switches to a prosurvival mode, activates NOS3 and is un-acetylated at the Lys^118^ residue. Mechanistically we found that oxygenation inhibits the expression of TIP60 acetylase enzyme. We have shown that the oxygen-induced inhibition of TIP60 in the rat infarct myocardium and rat cardiomyocytes results in un-acetylation or lack of acetylation of p53 at the Lys^118^ residue. The oxygen-dependent regulation of TIP60 appears to serve as a molecular mechanism through which oxygen can regulate the transcriptional activity of p53. Although how oxygen can regulate TIP60 expression is unknown at this time, our results suggest that TIP60 expression is repressed, resulting in the lack of p53Lys^118^ acetylation and lack of p53-dependent apoptosis and initiation of p53-prosurvival pathway in cardiac tissue exposed to high oxygen concentrations. On the contrary, hypoxia in the myocardium promotes TIP60 expression resulting in hyper-acetylation at the p53Lys^118^ residue, which induces p53-dependent apoptosis in the infarct heart. We have presented a predictive model suggesting possible molecular mechanism which might allow oxygen to regulate p53 transcriptional ability (Fig 10). Alterations in TIP60 levels act as a switch for p53 to sense changes in oxygen levels and initiate the apoptotic or prosurvival pathways by regulation of p53Lys^118^ acetylation. The lack of p53Lys^118^ acetylation evoked by oxygenation of infarct myocardium affects the ability of p53 to bind to BAX promoter and instead p53 binds to NOS3 promoter and activates a p53-induced NOS3-dependent survival program in the infarct myocardium.

**Figure 11:**
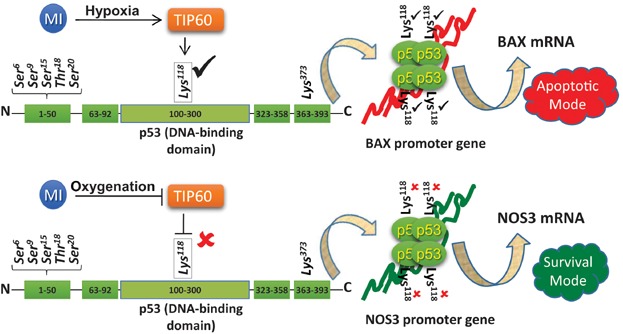
Schematic illustration of the molecular switch, which regulates the decision of p53 to activate BAX or NOS3 promoter.

**Figure 12:**
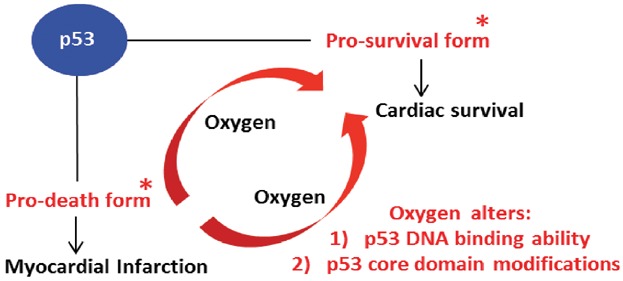
Schematic illustration of the model explaining the physiological relevance of the research work.

Previously, it was shown that p53-Lys^120^ acetylation is induced in response to DNA damage in cancer cells (Mellert et al, 2007; Tang et al, 2006). We have shown that conditions of serum deprivation and MI also activate TIP60-dependent p53-Lys^118^ acetylation. In cancer cells mutation of this lysine residue to an arginine abolished p53-mediated apoptosis but not cell-growth arrest (Tang et al, 2006). Conceptually, it was shown that this lysine site was responsible for the apoptotic effector response; however, mutation of this site did not kill the transcriptional activity of p53 protein but only altered it to another effector response of cell-cycle arrest. Our results show that p53-Lys^118^ mutated to Arg^118^ in the H9c2 cell and RNC does not inhibit the transcriptional activity of p53 but only targets its ability to activate its downstream apoptotic genes. Not only the mutation abolished p53-BAX-RE interaction but also it helped activation of p53-NOS3-RE in the oxygenated infarct hearts. This data suggest that the p53-Lys^118^ acetylation-dependent death or survival pathway in infarct and oxygenated hearts is conceptually similar to the observations of the p53-dependent apoptosis and its role in the survival of cancer cells. However, the mechanism through which oxygen regulates the expression of TIP60 protein is still unknown and requires further research. Based on the findings of the resent study, we have proposed a model to explain the molecular switch, which regulates the decision of p53 to activate BAX or NOS3 promoter (Fig 11). It is intriguing how oxygenation can bring about the changes in p53 protein allowing it to function as a pro-survival factor in the infarct myocardium. However, one must consider that p53 has inherent ability to choose between various pathways according to the cellular microenvironment it exists in. The decision of p53 choosing between death and survival and its role in cardioprotection in oxygenated infarct myocardium is a new chapter in p53 myriad. The physiological relevance of this mechanism is explained in as graphical representation (Fig 12). In future, cardioprotection and cardiac tissue regeneration might be achieved by generating p53-prosurvival form through inhibition of p53 core-domain post-translational modifications.

The present study provides a novel mechanistic insight and therapeutic strategy to target the infarction-induced myocyte apoptosis in the heart. The results have important biomedical and physiological relevance in the treatment of MI. Oxygen therapy is expected to improve the oxygenation of the ischemic myocardium, reduce infarct size, and consequently morbidity and mortality. Although the use of supplemental oxygen in the treatment of acute MI has been in practice for over 100 years, there is no conclusive data on its beneficial effect (Beasley et al, 2007; Wijesinghe et al, 2009). Controversies continue to emerge regarding the applicability and efficacy of oxygen therapy for MI patients (Kones, 2011). One-time administration of hyperoxygenation, intended as a pre-conditioning treatment before induction of myocardial injury, has been shown to be beneficial (Cabigas et al, 2006; Yogaratnam et al, 2008, 2010). However, these studies lacked the clinical relevance for treating post-MI patients. On the other hand, clinical protocols routinely use inhalation of high-flow oxygen in the first 24 h after acute MI. These clinical studies provided conflicting results, even detrimental effects, largely attributed to vasoconstrictive effect of oxygen (Kones, 2011; Wijesinghe et al, 2009). Our study provides a post-MI approach with daily cycles of brief periods of oxygenation, which is more practical and clinically relevant. Furthermore, the present study also provides the underlying molecular mechanism by which periodic administration of supplemental oxygenation results in pro-survival responses in the infarct heart.

## MATERIALS AND METHODS

### Cell culture

L6, H4TG and H9c2 cells were obtained from ATCC (USA) and were cultured as monolayers in DMEM medium supplemented with 10% (v/v) heat-inactivated foetal bovine serum and antibiotics, and incubated at 37°C in a humidified atmosphere of 95% air and 5% CO_2_. The RNC cells were procured from Lonza Walkersville, Maryland (#R-CM-561). The cells were cultured according to the supplier's protocol. The H9c2 p53^−/−^ cells were synthesized by knocking out p53 gene from H9c2 cells as described previously (Le Floch et al, 2011). The H9c2 p53-Lys^118^(Mut) cells were synthesized from H9c2 p53^−/−^ cells by stable transfection with the p53 cDNA coding for p53-Lys^118^ to Arg^118^ mutation as described previously (Gogna et al, 2012a). *Chemicals*; Resveratrol and pifithrin-alpha were purchased from Sigma (Saint Louis, MO, USA). *Antibodies*; Anti-p53, anti-actin, anti-GAPDH, anti-mdm2, anti-p300, anti-NOS3, anti-phospho-p53Ser^6^, anti-phospho-p53Ser^9^, anti-phospho-p53Ser^15^, anti-phospho-p53Ser^20^, anti-phospho-p53Thr^15^, anti-acetylated-p53Lys^120^, anti-acetylated-p53Lys^373^, anti-acetylated-p53Lys^379^, anti-BAX, anti-MOZ, anti-TIF2, anti-AIB1, anti-p300, anti-MOF, anti-RIP160, anti-CBP, anti-TIP60, anti-PCAF and anti-BRPF1 antibodies were purchased from Abcam. Anti-acetylated-p53Lys^120^ was purchased from Bethyl Laboratories (USA).

### Reporter construction and luciferase assay

A fragment spanning from −423 to −273 relative to the transcription start site of rat NOS3 genomic sequence (Accession no. GXP_1414475) was synthesized with *KPN*1 and *Hind*III sites (Sigma–Aldrich). This fragment was inserted into the *KPN*I and *Hind*III sites of pGL3-basic vector (Promega, Madison, WI, USA) to generate a NOS3 luciferase reporter (pGL3-NOS3). To generate pGL3-mtNOS3 plasmid, the p53 NOS3 binding site was mutated and the mutant DNA fragments were inserted into the pGL-3 vector. These recombinant plasmids were confirmed by DNA sequencing. Rat L6 cells were co-transfected with a luciferase reporter. The activity of reporters was evaluated with dual-Glo luciferase assay system (Promega). The levels of firefly luciferase activity were normalized to pRL-TK luciferase activity. The promoter region is as described here: (-423) TGAGCACTGG GCACATGGAC AGTGGGTGGT AGCTCCACCA GACCCCGCCT CCTCCCCAGC AAGCCCCATG CCAGCATGTC CTCTAGAGCT GATGGTCAAA ACCTCATCTC TTTTTTTCCT ACAACCTCGG CCGGTCCTCC TCGGACCTAG (-273). TIP60 siRNA and cDNA were purchased from Origene.

### Electrophoretic mobility shift assay

The p53 EMSA was performed as described (Hainaut & Milner, 1993). Fifty nanograms of purified protein was mixed with 9 µl of binding buffer (HEPES pH 7.6 20 mM, NaCl 10 mM, MgCl_2_ 1.5 mM, EDTA 0.2 mM, glycerol 20%, NP-40 0.1%, DTT 1 mM and PMSF 0.5 mM), 0.6 µl DTT, 2 µl salmon sperm, 1 µl BSA) and biotin-labelled probe. In order to increase the p53-specific DNA binding and to improve detection of the p53-specific band, 1 µl of the anti-p53 antibody 421 was added to each sample. The samples were then incubated for 30 min at room temperature and loaded on a 4% acrylamide gel. For super-shift experiments, 1 µl of the anti-p53 antibody 1801 (Oncogene Research Products, Cambridge, MA, USA) was added before incubation.

### Luciferase assay

Cells were plated in 35-mm petri dishes the day before transfection so that they reached 60–80% confluence upon transfection. Reporter plasmids (1.0–1.5 mg/well) were transfected with fugene transfection reagent (Qiagen) as per the manufacturer's instructions. After desired incubation period, the cells were washed in cold PBS three times and lysed with 200 ml of the lysis buffer by a freeze–thaw cycle, and lysates were collected by centrifugation at 14,000 rpm for 2 min in a bench-top centrifuge. Twenty microlitre of supernatant was used for the assay of luciferase activity using a kit (Promega) as per the manufacturer's instruction.

### Chromatin immunoprecipitation

The ChIP experiments were performed as described previously (Gogna et al, 2012a,2012c). Formaldehyde was added at a final concentration of 1% directly to cell culture media. Fixation proceeded at 22°C for 10 min and was stopped by the addition of glycine to a final concentration of 0.125 M. The cells were collected by centrifugation and rinsed in cold phosphate-buffered saline. The cell pellets were re-suspended in swelling buffer (10 mM potassium acetate, 15 mM magnesium acetate, 0.1 M Tris; pH 7.6, 0.5 mM phenylmethylsulfonyl fluoride, and 100 ng of leupeptin and aprotinin/ml), incubated on ice for 20 min, and then dounce-homogenized. The nuclei were collected by micro-centrifugation and then resuspended in sonication buffer (1% sodium dodecyl sulfate, 10 mM EDTA, 50 mM Tris–HCl; pH 8.1, 0.5 mM phenylmethylsulfonyl fluoride, and 100 ng of leupeptin and aprotinin/ml) and incubated on ice for 10 min. Prior to sonication, 0.1 g of glass beads (212- to 300-µm diameter; Sigma) was added to each sample. The samples were sonicated on ice with an ultrasonics sonicator at setting 10 for six 20-s pulses to an average length of approximately 1,000 bp and then micro-centrifuged. The chromatin solution was pre-cleared with the addition of *Staphylococcus aureus*. Prior to the first wash, 20% of the supernatant from the reaction with no primary antibody for each time point was saved as total input chromatin and was processed with the eluted immunoprecipitates beginning at the cross-link reversal step. Cross-links were reversed by the addition of NaCl to a final concentration of 200 mM, and RNA was removed by the addition of 10 µg of RNase A per sample followed by incubation at 65°C for 4–5 h. The samples were then precipitated at 20°C overnight by the addition of 2.5 volumes of ethanol and then pelleted by micro-centrifugation. The samples were re-suspended in 100 µl of Tris–EDTA; pH 7.5, 25 µl of 5× proteinase K buffer (1.25% sodium dodecyl sulfate, 50 mM Tris; pH 7.5, and 25 mM EDTA), and 1.5 µl of proteinase K (Boehringer Mannheim) and incubated at 45°C for 2 h. Samples were extracted with phenol–chloroform–isoamyl alcohol (25:24:1) followed by extraction with chloroform–isoamyl alcohol and then precipitated with 1/10 volume of 3 M NaOAc (pH 5.3), 5 µg of glycogen, and 2.5 volumes of ethanol. The pellets were collected by micro-centrifugation, resuspended in 30 µl of water, and analysed by PCR.

**The paper explained**

PROBLEM:

Myocardial infarction (MI) is a major form of cardiovascular disease and is the leading cause of death in patients. Myocardial infarction-related deaths are predicted to increase to 14% of all deaths globally in 2030. Typically in myocardial infarction, occlusion of coronary arteries by atherosclerosis results in decreased blood flow (ischemia) and oxygen deprivation in the myocardium and promotes apoptotic death of cardiomyocytes and impaired cardiac function. p53, the guardian of genome and regulatory master switch of cell death and proliferation, is active in ischemic and infarct hearts. p53-dependent apoptosis is a major player in the death of cardiomyocytes leading to left-ventricle (LV) remodelling and congestive heart failure. There is a great need to develop therapeutic interventions, which will reduce the p53-mediated cardiomyocyte apoptosis in MI patients. Recently, we established that supplemental oxygen therapy, administered in the form of brief cycles of hyperoxygenation, improved cardiac function by limiting cardiomyocyte apoptosis. We have also reported recently that cellular oxygen plays a crucial role in the regulation of p53-post translational modifications, p53-DNA interactions in the chromatin and p53-dependent transcription. In the present study, we studied the underlying molecular mechanism by which periodic administration of supplemental oxygenation results in alterations of p53-DNA interactions and p53 transcriptional ability and activation of a pro-survival p53 response in the infarct heart.

RESULTS:

Oxygen therapy of infarct hearts resulted in increased survival of cardiomyocytes, inhibition of p53-dependent apoptosis, and high expression of NOS3 gene via p53-dependnet transcription and improved cardiac function. The oxygenation of infarct hearts abolished the initiation of p53-dependent pro-apoptotic response and instead regulated p53 to initiate a pro-survival response in the infarct heart. The increased oxygen in the infarct heart modulated the post-translational profile and transcriptional activity of p53, in order to generate a pro-survival p53 type. Oxygenation led to a significant reduction in the cellular expression of TIP60, a p53 acetylase. Decrease in TIP60 expression in oxygenated infarct heart resulted in a p53 form which was phosphorylated and acetylated at other critical domains, except acetylation of p53-Lys^118^ residue. The results showed that acetylation of p53-Lys^118^ residue acts as a molecular switch in determining the ability of p53 to function as an apoptotic or an anti-apoptotic protein. The p53-Lys^118^ acetylated form showed high affinity for p53 binding site at the BAX promoter and activated a pro-apoptotic program in the infarct heart and cardiomyocytes. On the other hand, the oxygen therapy-induced p53-Lys^118^ non-acetylated form in infarct heart showed a high affinity towards the p53 binding site in the NOS3 promoter and initiated a p53-dependent pro-survival response.

IMPACT:

This research work provides a novel mechanistic insight (pathway) and therapeutic strategy to target the infarction-induced apoptosis in the myocardium. Further, the decision between p53-dependent apoptosis or the p53-dependent pro-survival response in the infarct myocardium lies between the acetylated and non-acetylated p53-Lys^118^ forms. Thus, molecular strategies which will be designed to de-acetylate p53 at Lys^118^ residue will provide therapeutic benefits to patients suffering from myocardial infarction. As advance of basic science knowledge, the research work shows the importance cellular oxygenation in the regulation of post-translational modifications and DNA-protein interactions. In addition, the research work shows the ability of p53 to function as a cardioprotective protein by initiating a NOS3-dependnet pro-survival response in the oxygen-treated infarct myocardium.

### Putative transcription-factor-binding-site (TFBS) analysis on NOS3 promoter

TFBSs specific to *p53* were analysed in NOS3 promoter sequence (−900/+150 bp) using MatInspector (Genomatix, Munich, Germany) with MatBase matrix library 8.0. MatInspector can be used online at http://www.genomatix.de/en/index.html and details regarding the weight matrices used to identify potential TFBSs have been described previously (Cartharius et al, 2005). The p53 binding site was identified between −340 and −363 base pairs from the transcription start site. This binding site had homology score of 0.92 for consensus p53 DNA binding site.

### Gene array

The RT^2^ Profiler PCR Array kit PAHS-027z and PAHS-012z were used to profile the expression of key genes involved in programmed cell death as described previously (Gogna et al, 2012a).

### Statistical analysis

Data were expressed as mean ± SD (standard deviation). Differences between two groups were tested using unpaired Student's *t*-test using SigmaStat software. A *p*-value of <0.05 was considered as statistically significant.

## Supplementary material

As a service to our authors and readers, this journal provides supporting information supplied by the authors. Such materials are peer reviewed and may be re-organized for online delivery, but are not copy-edited or typeset. Technical support issues arising from supporting information (other than missing files) should be addressed to the authors.

**Figure S1.** Purity check on the nuclear and cytoplasmic fractions. Immunoprecipitation (IP) with anti-PARP (nuclear protein) and anti-tubulin (cytoplasmic protein) antibodies was conducted in control (healthy) heart tissues to check the purity of the nuclear (NF) and cytoplasmic (CF) fractions. The images (representative of *n* = 4) show that the nuclear and cytoplasmic fractions are pure as evidenced by the lack of tubulin protein in the nuclear fraction and lack of PARP in the cytoplasmic fraction.

**Figure S2.** Diagrammatic representation of pNOS3p-luc1 vector construct. The 602 base-pair NOS3 promoter, upstream of the transcription start site is shown.

**Figure S3.** Effect of p53 on NOS3 promoter. pNOS3p-luc1 was transfected into H4TG hepatoma cells. Resveratrol was used to activate p53 transcription, which induced a 12-fold increase in pNOS3p-luc1 in resveratrol-treated H4TG cells. p53 gene-silencing using p53 siRNA abolished the activation of p53 promoter. Data represent mean ± SD of eight independent measurements. **p* = 2.1E−09.

**Figure S4.** Role of p53 in the upregulation of NOS3 gene in L6 cells. The cells were treated with 10-µM resveratrol to activate p53. Results show a marked increase in NOS3 mRNA and protein expression in the resveratrol-treated cells. Upon p53 gene-silencing using p53 siRNA, the resveratrol-mediated increase in NOS3 gene was abolished, suggesting a role for NOS3 in regulation of NOS3 gene.

**Figure S5.** Diagrammatic representation of the minimal pNOS3p-luc2 vector construct. pNOS3p-luc2 (NOS3-p53RE) and the mmpNOS3p-luc2 (mutant minimal promoter2, with mutated p53RE sequence) vector constructs are shown.

**Figure S6.** Co-localization of p53 and NOS3 proteins in the infarct heart tissue. Immunohistochemical staining of NOS3 and p53 were performed using NOS3 and p53 antibodies. The fluorescence microscopic images of DAPI (nuclear staining), p53 and NOS3 are superimposed to show their co-localization in the healthy (control), infarct (MI) and oxygen-treated (MI + OxCy) hearts.

**Figure S7.** Binding of p53 protein on the BAX gene promoter. Chromatin immunoprecipitation (ChIP) was used to analyse the binding of p53 protein on the BAX gene promoter in the MI and MI + OxCy treated cardiac tissue. Results show that p53 does not bind to the BAX promoter in the healthy hearts, however strong p53 binding to the BAX promoter was observed in the MI heart tissue (lane 3). MI + SC hearts serve as controls (lane 4). p53 does not bind to BAX promoter in the MI + OxCy cardiac tissue (lane 5).

**Figure S8.** BAX mRNA level in the infarct heart tissue. BAX mRNA levels in MI and treated hearts were measured using RT-PCR. Results show that BAX mRNA is upregulated in the MI hearts. MI hearts treated with mesenchymal stem cell (MI + MSC), oxygenation (MI + OxCy) or combination (MI + MSC + OxCy) abolished BAX mRNA synthesis.

**Figure S9.** BAX protein level in the infarct heart tissue. BAX protein levels in MI and treated hearts were measured using Western blotting. Results show that BAX protein is upregulated in the MI heart. MI hearts treated with mesenchymal stem cell (MI + MSC), oxygenation (MI + OxCy) or combination (MI + MSC + OxCy) abolished BAX protein.

**Figure S10.** *In vivo* ELISA of p53 modifications. Phosphorylation and acetylation of p53 at the known sites were quantified in the healthy (control), MI and the MI + OxCy hearts. The results show significant increase in p53 phosphorylation of acetylation in the MI hearts in comparison to the control hearts. Interestingly the acetylation of p53Lys118 residue is significantly decreased upon oxygenation of the MI hearts. Data represent mean ± SD of seven independent measurements.
