# Supplementary material for: p53's choice of myocardial death or survival: Oxygen protects infarct myocardium by recruiting p53 on NOS3 promoter through regulation of p53-Lys118 acetylation
Source: EMBO Mol Med. 2013 Oct 1;5(11):1662–83. doi: 10.1002/emmm.201202055 (PMC3840484; doi:10.1002/emmm.201202055)
Supplement: Supplementary file 1 [file emmm0005-1662-SD1.pdf]

EMBO Molecular Medicine

p53's choice of myocardial death or survival: Oxygen protects infarct myocardium by recruiting p53 on NOS3 promoter through regulation of p53-Lys118 acetylation

Rajan Gogna, Esha Madan, Mahmood Khan, Uttam Pati and Periannan Kuppusamy

Corresponding author: Periannan Kuppusamy, Dartmouth College

Review timeline:

Submission date:	06 August 2013
Editorial Decision:	20 November 2013
Revision received:	24 January 2013
Editorial Decision:	13 February 2013
Revision received:	10 July 2013
Editorial Decision:	18 July 2013
Revision received:	26 July 2013
Editorial Decision:	30 July 2013
Revision received:	06 August 2013
Accepted:	09 August 2013

Transaction Report:

(Note: With the exception of the correction of typographical or spelling errors that could be a source of ambiguity, letters and reports are not edited. The original formatting of letters and referee reports may not be reflected in this compilation.)

Editor: Roberto Buccione

1st Editorial Decision 20 November 2013

Thank you for the submission of your manuscript " p53's choice of myocardial death or survival: Oxygen protects infarct myocardium by recruiting p53 on NOS3 promoter through regulation of p53-Lys118 acetylation".

We are very sorry for the delay in getting back to you with the Reviewers' evaluation on your work. Unfortunately, in this case we experienced unusual difficulties in securing three appropriate reviewers in a timely manner. We have now heard back from the three referees whom we asked to evaluate your manuscript.

You will see that although Reviewers 2 and 3 underline the considerable potential interest of your work, together with Reviewer 1, they also raise significant concerns that prevent us from considering publication at this time.

Reviewer 1 is critical of the experimental approaches, choice of cell model and the interpretations/conclusions drawn from your data. S/he complains the lack of histology data to support p53/eNOS localisation during myocardial remodelling and challenges the use of H9c2 cells to explore p53 function in cardiomyocytes.

Reviewer 2 is supportive of your study, but mentions a number of specific issues that weaken your

current claims. For instance, s/he points to the need of qPCR to validate RT-PCR data, ELISA to quantify the effects of oxygen on p53 in hearts and the general lack of controls in immunoprecipitation, co-immunoprecipitation and other experiments.

Reviewer 3, while also being generally supportive, suggests some improvements to increase flow, readability and discussion of the medical implications of your study.

While it is clear that publication of the paper cannot be considered at this stage, I am open to the submission of a substantially revised manuscript, provided, however, that the Reviewers' concerns are fully addressed with additional experimental data where appropriate.

Please note that it is EMBO Molecular Medicine policy to allow a single round of revision only and that, therefore, acceptance or rejection of the manuscript will depend on the completeness of your responses included in the next, final version of the manuscript.

As you know, EMBO Molecular Medicine has a "scooping protection" policy, whereby similar findings that are published by others during review or revision are not a criterion for rejection. I understand that the amount of work that would be required to submit a revised version of your manuscript is significant and I would understand if you decided to submit your work elsewhere. If you should decide to submit a revised version, I do ask you to let us know and then to get in touch with us after three months if you have not completed it, to update us on the status. Please also contact us as soon as possible if similar work is published elsewhere.

***** Reviewer's comments *****

Referee #1 (Comments on Novelty/Model System):

NOS3 function is characterized in endothelial cells. It's function in primary cardiomyocytes is unclear. P53 functions in a cell type specific way. Whole organ lysates are the wrong way to go.

Referee #1 (General Remarks):

Oxygen dependent regulatory function of p53 related to acetylation status is very interesting. However the authors do not provide any in vivo relevant data. NOS3 functions are well characterized in endothelial cells. It's function in primary cardiomyocytes is unclear. P53 functions in a cell type specific way. Whole organ lysates are the wrong way to go.
There are no histology data showin co localization of p53/eNOS during myocardial remodeling. In vitro studies were performed in H9c2 which is not an adequate system to explore p53 function in cardiomyocytes. Most experiments are missing important controls. Data are over interpretated.

Referee #2 (Comments on Novelty/Model System):

Novelty: The concept of a change in acetylation of a single p53 lysine altering p53 preference for one promoter or another with significant functional effects is particularly novel and intriguing.

Technical quality: Technical strengths of the study include the use of a variety of approaches to test this hypothesis and the high quality of the data presented in the manuscript.

Medical relevance: The physiological and medical relevance of the work is high, since the authors show the role of a simple biological molecule such as oxygen in regulation of transcription, post-translational modifications and, most importantly, pro-survival responses in infarct myocardium.

I have no major problems with the adequacy of the model system.

Referee #2 (General Remarks):

The manuscript by Gogna et al describes an interesting research work about how oxygen can switch p53 signaling from a pro-death to pro-survival program by preventing acetylation of Lys118 residue. Myocardial infarction causes oxygen deficiency in tissue and induces p53-mediated cell-death through the BAX apoptosis pathway while oxygenation of the infarct heart can prevent cell-death and tissue damage. The concept of a change in acetylation of a single p53 lysine altering p53 preference for one promoter or another with significant functional effects is intriguing. The study shows that oxygenation can shift p53 binding from BAX-p53RE to NOS3-p53RE, which upregulates NOS3 expression to promote cell survival. Low p53 Lys118 acetylation by the oxygen-mediated reduction of TIP60 acetylase is shown to play a role by switching the binding of p53 from BAX-p53RE to NOS3-p53RE. This prevents the expression of various cell-death proteins while inducing expression of multiple anti-apoptotic genes. The research work is well organized and strengths of the study include the use of a variety of approaches to test this hypothesis and the high quality of the data presented in the manuscript. The results obtained support the conclusions and the physiological relevance of the work is high, since the authors show the role of a simple biological molecule such as oxygen in regulation of transcription, post-translational modifications and pro-survival responses in infarct myocardium. Although the manuscript is well organized authors have missed some important experiments and addition of the following experiments will further improve the quality of the data.
1) In Fig 1a the results of the RT-PCR must be counter validated and quantified using real time PCR.
2) In Fig 1b authors must include techniques like ELISA to quantify the effect of oxygen on p53 expression in infarct and treated hearts.
3) In Fig 1c the IP experiments must have a negative control such as IP without any antibody and with an unrelated antibody.
4) In Fig1d the authors must provide the controls in the supplementary data for obtaining the true nuclear and cytoplasmic fractions from the tissue samples.
5) Fig 1f the results of co-IP must be repeated with both p53 and p300 antibodies, currently the authors have shown the IP data with only p53 Ab.
6) In Fig 2b the blot must include a negative control such as NOS3 siRNA.
7) In Fig 2b and 2c the significance of the differences in the luciferase reading must be shown.
8) In Fig 2d the authors mush show the super-shift of the p53-DNA complex and add p21 p53RE as a positive control.
9) In Fig 3d authors should represent the binding of p53 to both bax-p53RE and NOS3-p53 RE in one EMSA experiment.
10) In Fig 4a the authors must present western blot for individual post translational modifications. The IP data must be quantified with protein quantification techniques such as ELISA.
11) In Fig 5c and 5d the significance of the results must be represented.

Referee #3 (Comments on Novelty/Model System):

Much more consideration shold be given to the medical consequences of this work. This is the weak part of this paper

Referee #3 (General Remarks):

This paper is interesting because it tackles a problem of fundamental basic biology which is of interest i.e. the role of p53 in cardioprotection under conditions of enhanced oxygenation in the myocardium.
A strong point is the molecular methodology, which in my view is impeccable.
The weak points deal mainly with the bio-MEDICAL implications of this study
Major points I find:
1.- It is well-known that necrosis plays a MAJOR role in cell death in myocardial infarction (see for instance: "The injurious impact of myocardial ischaemia comes from a mixture of pro-apoptotic and necrosis-promoting signal Cardiovasc Res (2000) 45 (3): 630-641". The word (or the concept) "necrosis" does not appear in the paper.

2.- The introduction is far too long. One sometimes is misled by some thoughts that are not required for the understanding of this paper
3.- Please make an explanatory figure of how p53 SENSES oxygen.This is a strong statement in the paper and needs clarification (see also point 5) To this reviewer's knowledge, the mechanisms by which HIF senses oxygen are much clearer.
4.- Please comment on the lack of effectiveness of preconditioning in myocardial infarction treatment (clinical trials have so far failed to report positive effects in humans)
5.- Please make the paper easier to read. (For instance, too many abbreviations)

1st Revision - authors' response

Reviewer #1

R1.1 NOS3 function is characterized in endothelial cells. Its function in primary cardiomyocytes is unclear.

We agree with the reviewer that NOS3 is well-characterized in endothelial cells. However, cardiomyocytes also express NOS3 enzyme. Studies have demonstrated that NO production from cardiomyocyte-resident NOS3 is necessary for postnatal cardiomyocyte proliferation and maturation (Lepic et al, 2006). Both, iNOS and NOS3 (eNOS) isoforms are prominently expressed during early stages of cardiomyogenesis (Bloch et al, 1999). Studies have also shown that deficiency in NOS3 results in congenital atrial and ventricular septal defects (Feng et al, 2002).

R1.2 p53 functions in a cell type specific way. Whole organ lysates are the wrong way to go.

It's true that p53 functions in a cell-type specific way but study of p53 expression in the whole organs which consist of cells of a variety of different lineages such as brain, liver, prostrate, lungs, colon, testis, ovaries are conducted in past (Chresta et al, 1996; Iggo et al, 1990; Zhen et al, 1999). The prime reason for this is that the biomedical or physiological importance of the effect of p53 on whole organs holds a critical importance and further it is very hard and virtually non-practical to isolate cells of different origin from a tissue for all research studies. Thus the major physiological and biomedical relevance of the study lies in the observation, which shows the effect of oxygenation and p53-survival pathway in the heart as an organ, rather than individual cells of some lineage. In this study we have also used serum-deprived H9c2 cardiomyoblasts (Bonavita et al, 2003) to study the function of p53 in myocytes. We have observed similar results with respect p53 activation and function suggesting that p53's functions are not different in the tissue lysate containing cardiomyocytes and other cells.

R1.3 Oxygen dependent regulatory function of p53 related to acetylation status is very interesting. However the authors do not provide any in vivo relevant data. NOS3 functions are well characterized in endothelial cells. Its function in primary cardiomyocytes is unclear. P53 functions in a cell type specific way. Whole organ lysates are the wrong way to go.

Please see our response to R1.1 & R1.2, above.

R1.4 There are no histology data showing co-localization of p53/eNOS during myocardial remodeling.

As the reviewer suggested we have provided histology data showing co-localization of p53 and eNOS (NOS3) during myocardial remodeling. The new data is presented in Supplemental Figure 6 in the revised version.

R1.5 In vitro studies were performed in H9c2 which is not an adequate system to explore p53 function in cardiomyocytes.

H9c2 is a permanent cell line derived from embryonic rat heart tissue. H9c2 cells show electrophysiological and biochemical properties of both skeletal and cardiac tissues. The H9c2 cells have emerged as an excellent in vitro alternative to primary cardiac myocytes. The cells can be engineered to express foreign genes at controllable levels, making them a suitable system to study molecular responses to oxidative damage. Watkins et al (Watkins et al, 2011) showed the importance of H9c2 cells as a model for in vitro studies of cardiac hypertrophy and similarity with human cardiomyocyte cell lines for prospective molecular studies in heart development and disease. H9c2 cells showed almost identical hypertrophic responses to those observed in primary

© EMBO

cardiomyocytes. Although the H9c2 cells lack the elaborate contractile apparatus of bona fide cardiac myocytes, they cells elicit robust hypertrophy-associated signature of fetal gene expression. Additionally, similar to what occurs in the intact heart, pathological hypertrophy of H9c2 cardiac myocytes could be attenuated by pan-HDAC inhibitors.

R1.6 Most experiments are missing important controls. Data are over interpreted.

We have thoroughly revised our manuscript, and added appropriate controls as suggested by this, as well as by other reviewers. Further, we have simplified data interpretation using an additional model in the discussion section (Figure 8).

Reviewer #2

The manuscript by Gogna et al describes an interesting research work about how oxygen can switch p53 signaling from a pro-death to pro-survival program by preventing acetylation of Lys118 residue. Although the manuscript is well organized authors have missed some important experiments and addition of the following experiments will further improve the quality of the data.

R2.1 In Fig 1a the results of the RT-PCR must be counter validated and quantified using real time PCR.

As the reviewer suggested we have added qPCR data in Figure 1 (see Fig 1b in the revised manuscript).

R2.2 In Fig 1b authors must include techniques like ELISA to quantify the effect of oxygen on p53 expression in infarct and treated hearts.

As suggested, we have added *in vivo* ELISA data in Figure 1 (see Fig 1d in the revised manuscript).

R2.3 In Fig 1c the IP experiments must have a negative control such as IP without any antibody and with an unrelated antibody.

As the reviewer suggested, we have repeated the IP experiment with negative controls, IP without any antibody, IP with anti-tubulin antibody. The new data is presented in Fig 1e in the revised manuscript.

R2.4 In Fig 1d the authors must provide the controls in the supplementary data for obtaining the true nuclear and cytoplasmic fractions from the tissue samples.

As the reviewer suggested, we have provided an extra figure (Figure S1 in the revised manuscript) showing controls for obtaining true nuclear (NF) and cytoplasmic (CF) fractions.

R2.5 In Fig 1f the results of co-IP must be repeated with both p53 and p300 antibodies, currently the authors have shown the IP data with only p53 Ab.

As the reviewer suggested, we have repeated the analysis and added results showing co-IP with both p53 and p300 antibodies (see Fig 1h in the revised manuscript).

R2.6 In Fig 2b the blot must include a negative control such as NOS3 siRNA.

As the reviewer suggested, we have added NOS3 siRNA as a negative control in Figure 2b

R2.7 In Fig 2b and 2c the significance of the differences in the luciferase reading must be shown.

We have now mentioned the significance (P value) of results in figure 2b/2c legends.

R2.8 In Fig 2d the authors mush show the super-shift of the p53-DNA complex and add p21 p53RE as a positive control.

As the reviewer suggested, we repeated this experiment and added the lane showing super-shift of the p53-DNA complex and also we have added p21p53RE-p53 interaction lane as positive control in this experiment. The new data is shown in Fig 2d in the revised manuscript.

R2.9 In Fig 3d authors should represent the binding of p53 to both bax-p53RE and NOS3-p53 RE in one EMSA experiment.

As the reviewer suggested, we have repeated this experiment and we have now shown the binding of Bax-p53RE and NOS3-p53 RE in one EMSA experiment (Fig 3d).

R2.10 In Fig 4a the authors must present western blot for individual post-translational modifications. The IP data must be quantified with protein quantification techniques such as ELISA.

As the reviewer suggested, we have added additional controls in the immunoprecipitation data. Further, we have repeated the experiment using *in vivo* ELISA and the new results are presented in Figure S10.

R2.11 In Fig 5c and 5d the significance of the results must be represented.

We have now mentioned the significance and P values in figure 5c and 5d and in figure legends

Reviewer #3

R3.0 Much more consideration should be given to the medical consequences of this work. This is the weak part of this paper.

We have discussed the bio-medical implications of this research work as a separate section in the discussion. The following is a reproduction of the new text added at the end of Discussion:

"The present study provides a novel mechanistic insight and therapeutic strategy to target the infarction-induced myocyte apoptosis in the heart. The results have important biomedical and physiological relevance in the treatment of myocardial infarction. Oxygen therapy is expected to improve the oxygenation of the ischemic myocardium, reduce infarct size, and consequently morbidity and mortality. Although, the use of supplemental oxygen in the treatment of acute MI has been in practice for over 100 years, there is no conclusive data on its beneficial effect (Beasley et al, 2007; Wijesinghe et al, 2009). Controversies continue to emerge regarding the applicability and efficacy of oxygen therapy for MI patients (Kones, 2011). One-time administration of hyperoxygenation, intended as a pre-conditioning treatment before induction of myocardial injury, has been shown to be beneficial (Cabigas et al, 2006; Yogaratnam et al, 2008; Yogaratnam et al, 2010). However, these studies lacked the clinical relevance for treating post-MI patients. On the other hand, clinical protocols routinely use inhalation of high-flow oxygen in the first 24 hours after acute MI. These clinical studies provided conflicting results, even detrimental effects, largely attributed to vasoconstrictive effect of oxygen (Kones, 2011; Wijesinghe et al, 2009). Our study provides a post-MI approach with daily cycles of brief periods of oxygenation, which is more practical and clinically relevant. Furthermore, the present study also provides the underlying molecular mechanism by which periodic administration of supplemental oxygenation results in pro-survival responses in the infarct heart."

This paper is interesting because it tackles a problem of fundamental basic biology which is of interest i.e. the role of p53 in cardioprotection under conditions of enhanced oxygenation in the myocardium. A strong point is the molecular methodology, which in my view is impeccable.

We thank the reviewer for the recognition.

R3.1 The weak points deal mainly with the bio-MEDICAL implications of this study. Major points I find: It is well-known that necrosis plays a MAJOR role in cell death in myocardial infarction (see for instance: "The injurious impact of myocardial ischaemia comes from a mixture of pro-apoptotic and necrosis-promoting signal Cardiovasc Res (2000) 45 (3): 630-641". The word (or the concept) "necrosis" does not appear in the paper.

We have discussed the biomedical implications of our work in the revised paper. Please see our response to R3.0 above.

Both necrosis and apoptosis have been implicated in the loss (death) of cardiomyocytes in the reperfused heart (Kajstura et al, 1996; Yaoita et al, 2000). Studies using animal models of MI indicate that myocyte death due to necrosis is an early event that begins with prolonged ischemia, further exacerbated with the onset of reperfusion, and may last up to 24 hours (Eefting et al, 2004; Oerlemans et al, 2012). On the other hand, apoptotic cell death is largely initiated during reperfusion and continues to occur in the MI heart for longer duration (Gottlieb et al, 1994; Zhao et al, 2000). We used oxygenation treatment 48 hours after induction of MI by ischemia-reperfusion. The reason

for the 48-hour delay was to avoid introduction of hyperoxygenation during the onset of reactive oxidant production during the early phase of reperfusion, and subsequent inflammation.

We have added this information in the beginning of Introduction in the revised manuscript.

R3.2 The introduction is far too long. One sometimes is misled by some thoughts that are not required for the understanding of this paper.

As the reviewer suggested we have now shortened the introduction to 835 words from 1248 words in the previous version. We have also made the Introduction more streamlined and reader friendly.

R3.3 Please make an explanatory figure of how p53 SENSES oxygen. This is a strong statement in the paper and needs clarification (see also point 5). To this reviewer's knowledge, the mechanisms by which HIF senses oxygen are much clearer.

We have presented an extra model (Figure 8) in the discussion section which tries to explain a putative mechanism of oxygen sensing via a p53-TIP60 pathway.

R3.4 Please comment on the lack of effectiveness of preconditioning in myocardial infarction treatment (clinical trials have so far failed to report positive effects in humans).

As the reviewer suggested we have mentioned the important issue which deals with the lack of effectiveness of preconditioning in myocardial infarction treatment in the discussion section.

R3.5 Please make the paper easier to read. (For instance, too many abbreviations)

We have removed unnecessary abbreviations, simplified and shortened the paper.

REFERENCES

Beasley R, Aldington S, Weatherall M, Robinson G, McHaffie D (2007) Oxygen therapy in myocardial infarction: an historical perspective. *J R Soc Med* 100: 130-133

Bloch W, Fleischmann BK, Lorke DE, Andressen C, Hops B, Hescheler J, Addicks K (1999) Nitric oxide synthase expression and role during cardiomyogenesis. *Cardiovasc Res* 43: 675-684

Bonavita F, Stefanelli C, Giordano E, Columbaro M, Facchini A, Bonafe F, Caldarera CM, Guarnieri C (2003) H9c2 cardiac myoblasts undergo apoptosis in a model of ischemia consisting of serum deprivation and hypoxia: inhibition by PMA. *FEBS letters* 536: 85-91

Cabigas BP, Su J, Hutchins W, Shi Y, Schaefer RB, Recinos RF, Nilakantan V, Kindwall E, Niezgoda JA, Baker JE (2006) Hyperoxic and hyperbaric-induced cardioprotection: role of nitric oxide synthase 3. *Cardiovasc Res* 72: 143-151

Chresta CM, Masters JR, Hickman JA (1996) Hypersensitivity of human testicular tumors to etoposide-induced apoptosis is associated with functional p53 and a high Bax:Bcl-2 ratio. *Cancer research* 56: 1834-1841

Eefting F, Rensing B, Wigman J, Pannekoek WJ, Liu WM, Cramer MJ, Lips DJ, Doevendans PA (2004) Role of apoptosis in reperfusion injury. *Cardiovasc Res* 61: 414-426

Feng Q, Song W, Lu X, Hamilton JA, Lei M, Peng T, Yee SP (2002) Development of heart failure and congenital septal defects in mice lacking endothelial nitric oxide synthase. *Circulation* 106: 873-879

Gottlieb RA, Burleson KO, Kloner RA, Babior BM, Engler RL (1994) Reperfusion injury induces apoptosis in rabbit cardiomyocytes. *The Journal of clinical investigation* 94: 1621-1628

Iggo R, Gatter K, Bartek J, Lane D, Harris AL (1990) Increased expression of mutant forms of p53 oncogene in primary lung cancer. *Lancet* 335: 675-679

Kajstura J, Cheng W, Reiss K, Clark WA, Sonnenblick EH, Krajewski S, Reed JC, Olivetti G, Anversa P (1996) Apoptotic and necrotic myocyte cell deaths are independent contributing variables of infarct size in rats. *Lab Invest* 74: 86-107

Kones R (2011) Oxygen therapy for acute myocardial infarction-then and now. A century of uncertainty. *Am J Med* 124: 1000-1005

Lepic E, Burger D, Lu X, Song W, Feng Q (2006) Lack of endothelial nitric oxide synthase decreases cardiomyocyte proliferation and delays cardiac maturation. *Am J Physiol Cell Physiol* 291: C1240-1246

Oerlemans MI, Koudstaal S, Chamuleau SA, de Kleijn DP, Doevendans PA, Sluijter JP (2012) Targeting cell death in the reperfused heart: Pharmacological approaches for cardioprotection. *Int J Cardiol*

Watkins SJ, Borthwick GM, Arthur HM (2011) The H9C2 cell line and primary neonatal cardiomyocyte cells show similar hypertrophic responses in vitro. *In Vitro Cell Dev Biol Anim* 47: 125-131

Wijesinghe M, Perrin K, Ranchord A, Simmonds M, Weatherall M, Beasley R (2009) Routine use of oxygen in the treatment of myocardial infarction: systematic review. *Heart* 95: 198-202

Yaoita H, Ogawa K, Maehara K, Maruyama Y (2000) Apoptosis in relevant clinical situations: contribution of apoptosis in myocardial infarction. *Cardiovasc Res* 45: 630-641

Yogaratnam JZ, Laden G, Guvendik L, Cowen M, Cale A, Griffin S (2008) Pharmacological preconditioning with hyperbaric oxygen: can this therapy attenuate myocardial ischemic reperfusion injury and induce myocardial protection via nitric oxide? *J Surg Res* 149: 155-164

Yogaratnam JZ, Laden G, Guvendik L, Cowen M, Cale A, Griffin S (2010) Hyperbaric oxygen preconditioning improves myocardial function, reduces length of intensive care stay, and limits complications post coronary artery bypass graft surgery. *Cardiovasc Revasc Med* 11: 8-19

Zhao ZQ, Nakamura M, Wang NP, Wilcox JN, Shearer S, Ronson RS, Guyton RA, Vinten-Johansen J (2000) Reperfusion induces myocardial apoptotic cell death. *Cardiovasc Res* 45: 651-660

Zhen HN, Zhang X, Bu XY, Zhang ZW, Huang WJ, Zhang P, Liang JW, Wang XL (1999) Expression of the simian virus 40 large tumor antigen (Tag) and formation of Tag-p53 and Tag-pRb complexes in human brain tumors. *Cancer* 86: 2124-2132

2nd Editorial Decision 13 February 2013

Thank you for the submission of your revised manuscript to EMBO Molecular Medicine.

We have now heard back from the two Reviewers whom we asked to evaluate your manuscript. You will see that, while Reviewer 2 is satisfied with the revision, Reviewer 1 notes that his/her requests have not been addressed and thus does not support publication.

Reviewer 1 remains concerned that the clonally derived myoblast cell line H9c2 is not sufficient to draw conclusions on p53 function in cardiomyocytes. S/he correctly notes that neonatal rat cardiomyocytes are available and thus must be used to confirm key findings. Reviewer 1 had made this point clear in his/her first evaluation and furthermore, in my decision letter, I had asked you to comply with the Reviewers' requests. I thus concur with Reviewer 1's current assessment.

As you know, we would normally not allow a second revision. I am prepared in this case, however, to give you another opportunity to improve your manuscript, with the understanding that acceptance or rejection of the manuscript will depend the satisfactory validation of the key findings in cardiomyocytes in the next, final version of the manuscript.

As you know, EMBO Molecular Medicine has a "scooping protection" policy, whereby similar findings that are published by others during review or revision are not a criterion for rejection. However, I do ask you to get in touch with us after three months if you have not completed your revision, to update us on the status. Please also contact us as soon as possible if similar work is published elsewhere.

I look forward to seeing a revised form of your manuscript as soon as possible.

Should you find that the requested revisions are not feasible within the constraints outlined here and choose, therefore, to submit your paper elsewhere, we would welcome a message to this effect.

***** Reviewer's comments *****

Referee #1 (General Remarks):

The revised version of the manuscript by Gogna et al addressed several concerns but the main concerns were not addressed adequately.
The authors imply that NOS3 is an important modulator of p53 in cardiomyocytes and that this pathway is an important prosurvival mechanism for cardiomyocytes. My concerns are that the findings from whole organ lysates do not reflect signaling changes in cardiomyocytes during cardiac remodeling. In fact, stainings for NOS3 and p53 support these concerns. There is no expression of NOS3 and p53 in normal cardiomyocytes as shown. After injury there is dramatic upregulation of NOS3 and p53 in cell populations that do not appear to be related to cardiomyocytes. Any changes in the expression of these genes in whole organ lysates are probably related to signaling changes in non cardiomyocytes and through changes in cell populations (infiltration of inflammatory cells, massive death of other cells, proliferation of local non cardiomyocytes ect) during the remodeling process.
There is evidence in the literature that NOS3 is not expressed in cardiomyocytes (Tambascia et al Hypertension 2001; 37:1427-1428). The authors must show that p53 and NOS3 are co-expressed during the remodeling process.
The authors choose to use H9c2 cells which is a clonally derived myoblast lines that was generated over 40 years ago. We must assume that these cells bypass normal senescence through transformation affecting genetic stability. P53 is the gate keeper for genetic integrity. It is likely that p53 function is defective in H9s2 and therefore this line is less suitable to study p53 function. Neonatal rat cardiomyocytes are available and must be used at least to confirm key findings.

Referee #2 (Comments on Novelty/Model System):

Technical quality: The molecular biology and bio chemistry experimental designs, the technical correctness and data presentation is of the highest quality. All the important molecular biology and biochemistry tool have been used to reach the conclusions.
Novelty: The research brings about the novel role of oxygen in functioning as a cardioprotector by altering the transcriptional ability of p53.
Medical Impact: The use of oxygen to treat myocardial infraction will have huge bio-medical implications.
Adequacy of model system: The authors have used appropriate in-vivo model systems to conduct experiments and to reach the conclusions.

Referee #2 (General Remarks):

The revised version of the manuscript titled "p53's choice of myocardial death or survival: Oxygen protects infarct myocardium by recruiting p53 on NOS3 promoter through regulation of p53-Lys118 acetylation" now clearly establishes the role of oxygen as a modulator of p53's transcriptional ability to achieve cardioprotection via p53-NOS3 activation pathway contrary to the p53-BAX activation pathway which exists in the myocardial infarct hearts. The authors have addressed all my concerns related to addition of appropriate controls in the experiments and improving the overall quality of the manuscript. The paper now is suitable for publication in EMBO Molecular Medicine.

2nd Revision - authors' response 10 July 2013

Response to reviewer's comments

Editor

E: *Reviewer 1 remains concerned that the clonally derived myoblast cell line H9c2 is not sufficient to draw conclusions on p53 function in cardiomyocytes. S/he correctly notes that neonatal rat cardiomyocytes are available and thus must be used to confirm key findings. Reviewer 1 had made this point clear in his/her first evaluation and furthermore, in my decision letter, I had asked you to comply with the Reviewers' requests. I thus concur with Reviewer 1's current assessment. As you know, we would normally not allow a second revision. I am prepared in this case, however, to give you another opportunity to improve your manuscript, with the understanding that acceptance or rejection of the manuscript will depend the satisfactory validation of the key findings in cardiomyocytes in the next, final version of the manuscript.*

We thank the editor for giving us an opportunity to revise the manuscript. As suggested by the editor/reviewer 1, we have now performed the key experiments using rat neonatal cardiomyocytes. Accordingly, we have revised our manuscript and added the following new figures: Figure 6 (panel A-F), Figure 7 (panel B), Figure 9 (panel A-G). We have elucidated the whole molecular pathway in rat neonatal cardiomyocytes in addition to the H9C2 cells. The results observed with rat neonatal cardiomyocytes are similar to those in H9C2 cells and thus shows the critical role of oxygen in the regulation of p53 transcriptional activity.

Reviewer #1

R1.1 *The authors imply that NOS3 is an important modulator of p53 in cardiomyocytes and that this pathway is an important prosurvival mechanism for cardiomyocytes. My concerns are that the findings from whole organ lysates do not reflect signaling changes in cardiomyocytes during cardiac remodeling. In fact, stainings for NOS3 and p53 support these concerns. There is no expression of NOS3 and p53 in normal cardiomyocytes as shown. After injury there is dramatic upregulation of NOS3 and p53 in cell populations that do not appear to be related to cardiomyocytes. Any changes in the expression of these genes in whole organ lysates are probably related to signaling changes in non cardiomyocytes and through changes in cell populations (infiltration of inflammatory cells, massive death of other cells, proliferation of local non cardiomyocytes ect) during the remodeling process. There is evidence in the literature that NOS3 is not expressed in cardiomyocytes (Tambascia et al Hypertension 2001; 37:1427-1428). The authors must show that p53 and NOS3 are co-expressed during the remodeling process. The authors choose to use H9c2 cells which are a clonally derived myoblast lines that was generated over 40 years ago. We must assume that these cells bypass normal senescence through transformation affecting genetic stability.p53 is the gate keeper for genetic integrity. It is likely that p53 function is defective in H9c2 and therefore this line is less suitable to study p53 function. Neonatal rat cardiomyocytes are available and must be used at least to confirm key findings.*

We acknowledge the questions raised by the reviewer. As mentioned above, we have carefully addressed the concerns of the reviewer by repeating key experiments using rat neonatal cardiomyocytes (RNC). The new results confirm the results from H9C2 cells.
In our assessment the major concern of the reviewer originated from the uncertainty regarding the validity of the proposed study using H9C2 cells, which are clonally derived myoblast cells and not necessarily cardiomyocytes. The reviewer raised two major points: (1) Existence of eNOS or NOS3 as a signaling molecule in cardiomyocytes. (2) Suitability of H9C2 cells as basis of our *in-vitro* study model. We thank the reviewer for raising these questions and suggesting us to use rat neonatal cardiomyocytes.
In this revised version we have included the new results obtained using RNCs and compared with that obtained using H9C2 cells. We have validated the entire pathway in rat neonatal

cardiomyocytes. Our results with RNC support the previous findings with H9C2 cells where oxygenation inhibits the expression of TIP60 acetylase, resulting in the lack of p53 acetylation at Lys118 residue which promotes p53-NOS3 survival pathway. This phenomenon has now been observed by us in infarct hearts, H9C2 cells, and rat neonatal cardiomyocytes. We have added Figure 6a, 6b, 6c, 6d, 6e, 6f, 7b, 8a, 8b, 8c, 8d, 8e, 8f and 8g in support of our findings. We now sincerely hope that the reviewer will find that we have successfully addressed the concerns.

In addition, we would like to address the other concern of the reviewer that eNOS or NOS3 do not play any signaling role in the cardiomyocytes. In 2007, Barbara Casadei's research laboratory published the role of eNOS in cellular signaling in cardiomyocytes (Seddon et al., 2007). Using the model (shown below) the authors clearly showed the role of eNOS in signaling both in the coronary vasculature and in cardiomyocytes. In 2012, Allen Samarel and group from University of Chicago showed that all three isoforms are expressed in cardiomyocytes (Chu et al., 2012). They further showed that while eNOS and nNOS are constitutively expressed, iNOS is up-regulated under pathological conditions. These reports confirm our findings where we have discovered a very important role of molecular oxygen in inducing a p53-dependent survival pathway via NOS3 upregulation in the infarct hearts.

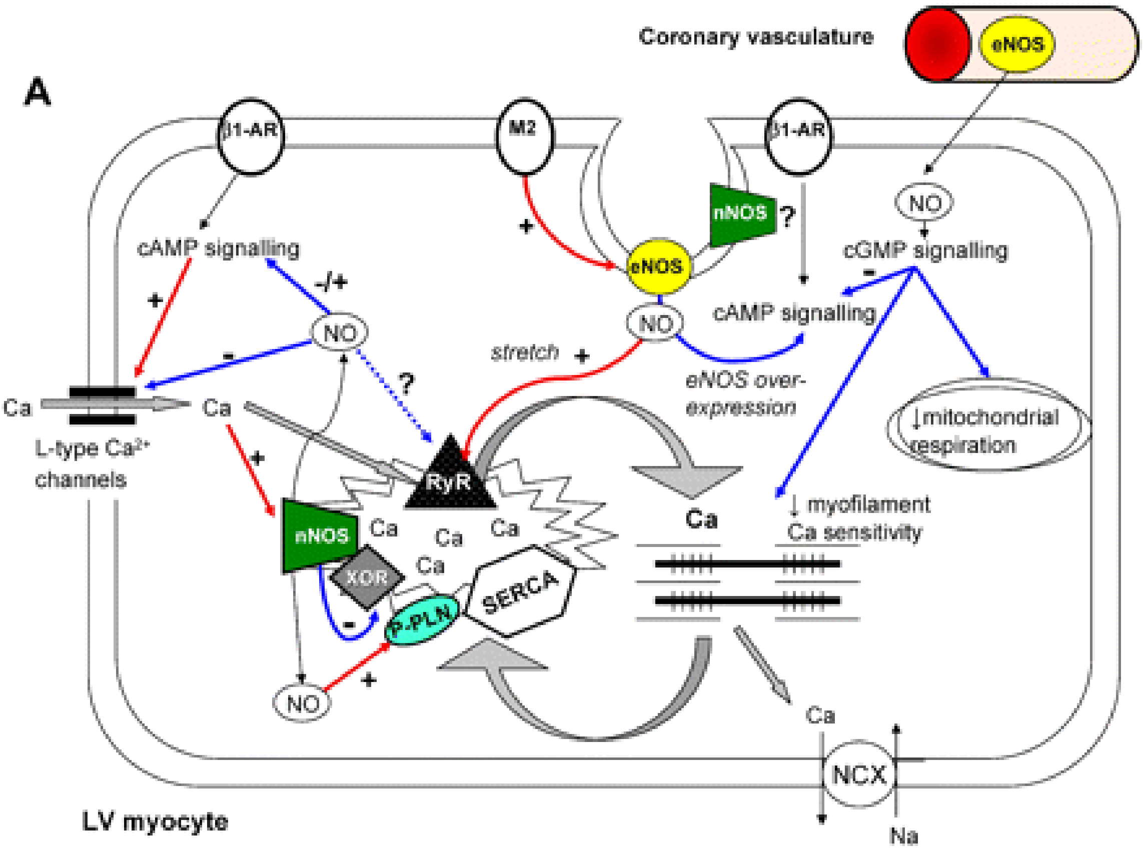

Cardiomyocytes as effectors of nitric oxide signaling. Mike Seddon et al, Cardiovascular Research 75 (2007) 315–326

References

Chu, M., Koshman, Y., Iyengar, R., Kim, T., Russell, B., and Samarel, A.M. (2012). Contractile Activity Regulates Inducible Nitric Oxide Synthase Expression and NO(i) Production in Cardiomyocytes via a FAK-Dependent Signaling Pathway. Journal of signal transduction *2012*, 473410.

Seddon, M., Shah, A.M., and Casadei, B. (2007). Cardiomyocytes as effectors of nitric oxide signalling. Cardiovascular Research *75*, 315-326.

3rd Editorial Decision 18 July 2013

Thank you for the submission of your revised manuscript to EMBO Molecular Medicine. We have now received the enclosed report from the Reviewer who was asked to re-assess it. As you will see the s/he is now globally supportive and I am pleased to inform you that we will be able to accept your manuscript pending the following final amendments:

As per our Author Guidelines, the description of all reported data that includes statistical testing must state the name of the statistical test used to generate error bars and P values, the number (n) of independent experiments underlying each data point (not replicate measures of one sample), and the actual P value for each test (not merely 'significant' or 'P < 0.05').

Please submit your revised manuscript within two weeks. Needless to say, the sooner we receive the final, revised version, the sooner I will be able to formally accept it for publication.

I look forward to reading a new revised version of your manuscript as soon as possible.

***** Reviewer's comments *****

Referee #1 (General Remarks):

The authors responded adequate to my comments.
No further comments or questions

3rd Revision - authors' response 26 July 2013

We are happy to know that our manuscript has been provisionally accepted for publication in EMBO Molecular Medicine.

As requested, we have included the p values, N numbers, and statistal method and software used in the calculations.

4th Editorial Decision 30 July 2013

Thank you for the submission of your revised manuscript to EMBO Molecular Medicine.

I am afraid that the manuscript is not yet acceptable for publiscation. In fact, the description of the reported data that includes statistical testing is stille incomplete. As I mentioned in my previous decision letter, the actual P value for each test (not merely 'significant' or 'P < 0.05') must be stated, in each and every case. This is missing in your revised manuscript for a numebr of figures and incompletely stated for others. Indeed, this version has even less details than the previous (V3) e.g. for figures 5, 6, 7, 8..

Please submit your correctly revised manuscript as soon as possible and in any case within two weeks.

I look forward to reading a new revised version of your manuscript as soon as possible.

We apologize for the lack of descriptive statistics. Since these p values were very low we used the lower limit, for example p<0.001 in our manuscript. We had removed the comparison I some cases as we thought it was not necessary. We apologize for the misunderstanding.

The "*" on Figure 8 is not needed as it is a single data. We have restored the notation (*) in Figure 7.

As requested, we have included the acctual p values, N numbers, details in the revised version of the manuscript.

We hope that this manuscript will now be suitable for formal acceptance and publication in EMBO Molecular Medicine.
