# Supplementary material for: p53's choice of myocardial death or survival: Oxygen protects infarct myocardium by recruiting p53 on NOS3 promoter through regulation of p53-Lys118 acetylation
Source: EMBO Mol Med. 2013 Oct 1;5(11):1662–83. doi: 10.1002/emmm.201202055 (PMC3840484; doi:10.1002/emmm.201202055)
Supplement: Supplementary file 2 [file emmm0005-1662-SD2.pdf]

SUPPLEMENTAL INFORMATION - TABLE OF CONTENT

1. SUPPLEMENTAL FIGURES AND LEGENDS 2

Figure S1. Purity check on the nuclear and cytoplasmic fractions 2

Figure S2. Diagrammatic representation of pNOS3p-luc1 vector construct 3

Figure S3. Effect of p53 on NOS3 promoter 4

Figure S4. Role of p53 in the upregulation of NOS3 gene in L6 cells.. 5

Figure S5. Diagrammatic representation of the minimal pNOS3p-luc2 vector construct............. 6

Figure S6. Co-localization of p53 and NOS3 proteins in the infarct heart tissue 7

Figure S7. Binding of p53 protein on the BAX gene promoter 8

Figure S8. BAX mRNA level in the infarct heart tissue 9

Figure S9. BAX protein level in the infarct heart tissue 10

Figure S10. In vivo ELISA of p53 modifications 11

2. SUPPLEMENTAL EXPERIMENTAL PROCEDURES.. 12

Western blotting 12

Preparation of nuclear and cytoplasmic extracts 12

Immunoprecipitation 12

Annexin-V staining 13

Protein ELISA 13

Induction of myocardial infarction 13

Protocol for oxygen-cycling 14

Immunohistochemical staining of cardiac tissue for p53 and nos3 expression 14

1. SUPPLEMENTAL FIGURES AND LEGENDS

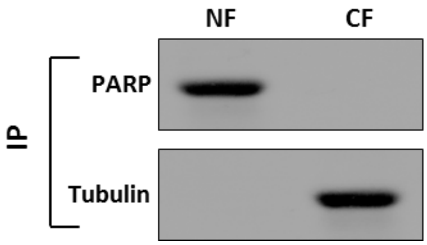

Figure S1. Purity check on the nuclear and cytoplasmic fractions. Immunoprecipitation (IP) with anti-PARP (nuclear protein) and anti-tubulin (cytoplasmic protein) antibodies was conducted in control (healthy) heart tissues to check the purity of the nuclear (NF) and cytoplasmic (CF) fractions. The images (representative of n=4) show that the nuclear and cytoplasmic fractions are pure as evidenced by the lack of tubulin protein in the nuclear fraction and lack of PARP in the cytoplasmic fraction.

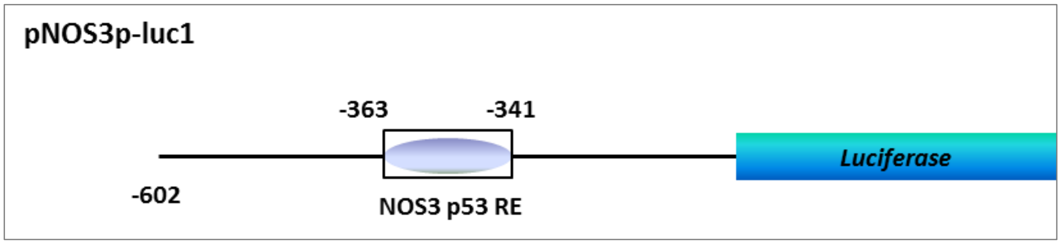

Figure S2. Diagrammatic representation of pNOS3p-luc1 vector construct. The 602 base-pair NOS3 promoter, upstream of the transcription start site is shown.

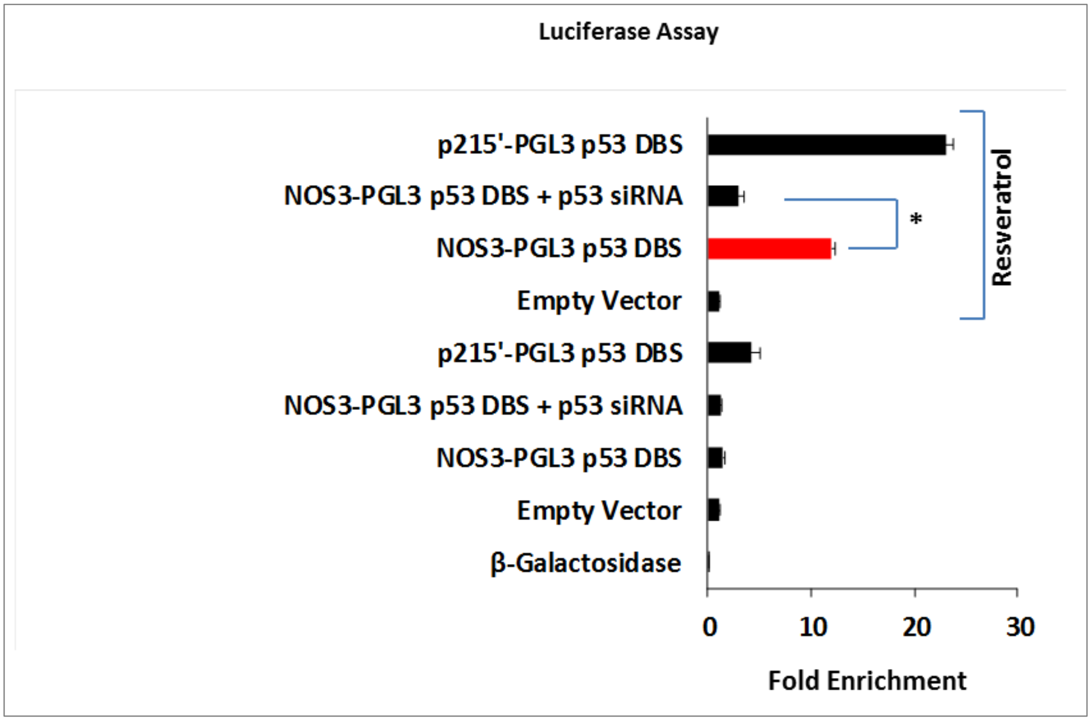

Figure S3. Effect of p53 on NOS3 promoter. pNOS3p-luc1 was transfected into H4TG hepatoma cells. Resveratrol was used to activate p53 transcription, which induced a 12-fold increase in pNOS3p-luc1 in resveratrol-treated H4TG cells. p53 gene-silencing using p53 siRNA abolished the activation of p53 promoter. Data represent mean±SD of 8 independent measurements. *p=2.1E-09.

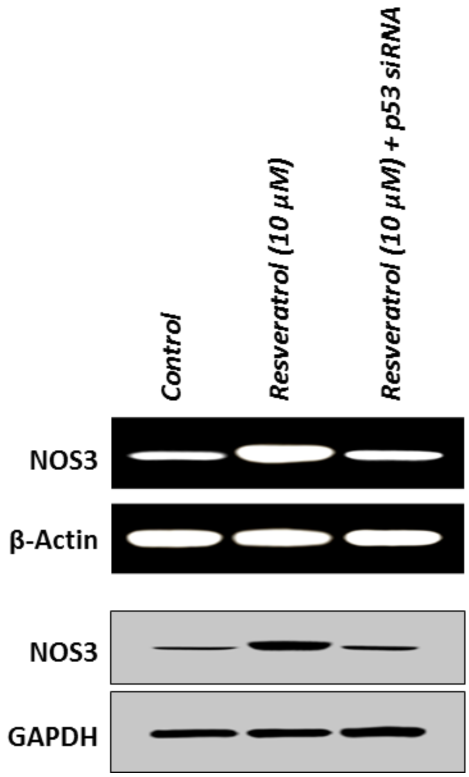

Figure S4. Role of p53 in the upregulation of NOS3 gene in L6 cells. The cells were treated with 10-μM resveratrol to activate p53. Results show a marked increase in NOS3 mRNA and protein expression in the resveratrol-treated cells. Upon p53 gene-silencing using p53 siRNA, the resveratrol-mediated increase in NOS3 gene was abolished, suggesting a role for NOS3 in regulation of NOS3 gene.

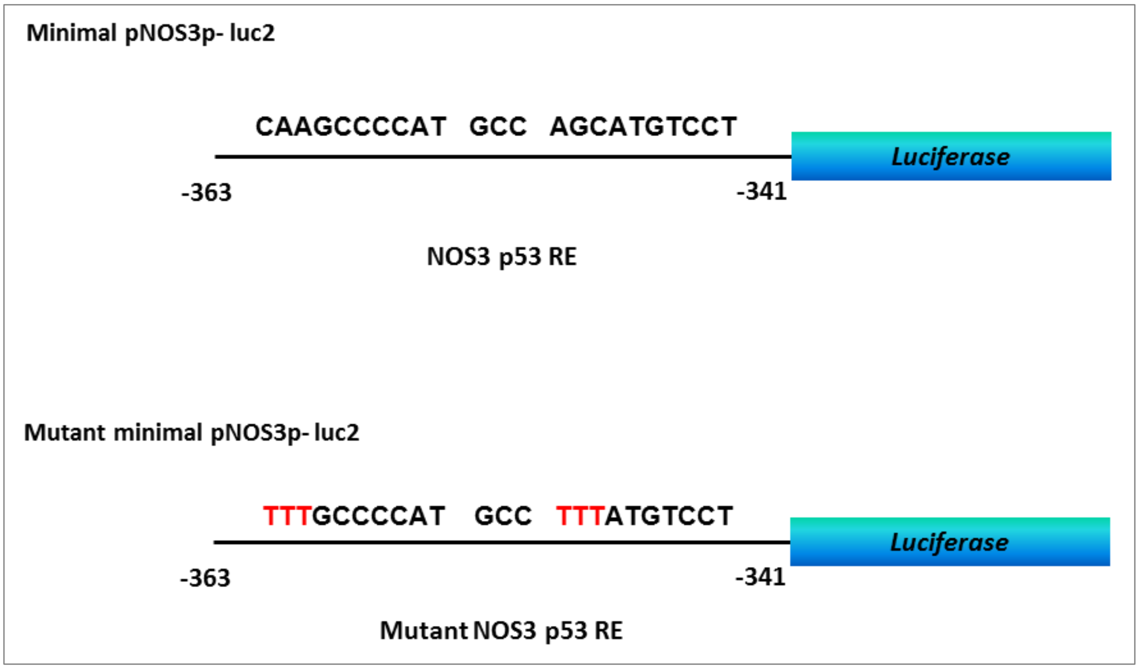

Figure S5. Diagrammatic representation of the minimal pNOS3p-luc2 vector construct. pNOS3p-luc2 (NOS3-p53RE) and the mmpNOS3p-luc2 (mutant minimal promoter2, with mutated p53RE sequence) vector constructs are shown.

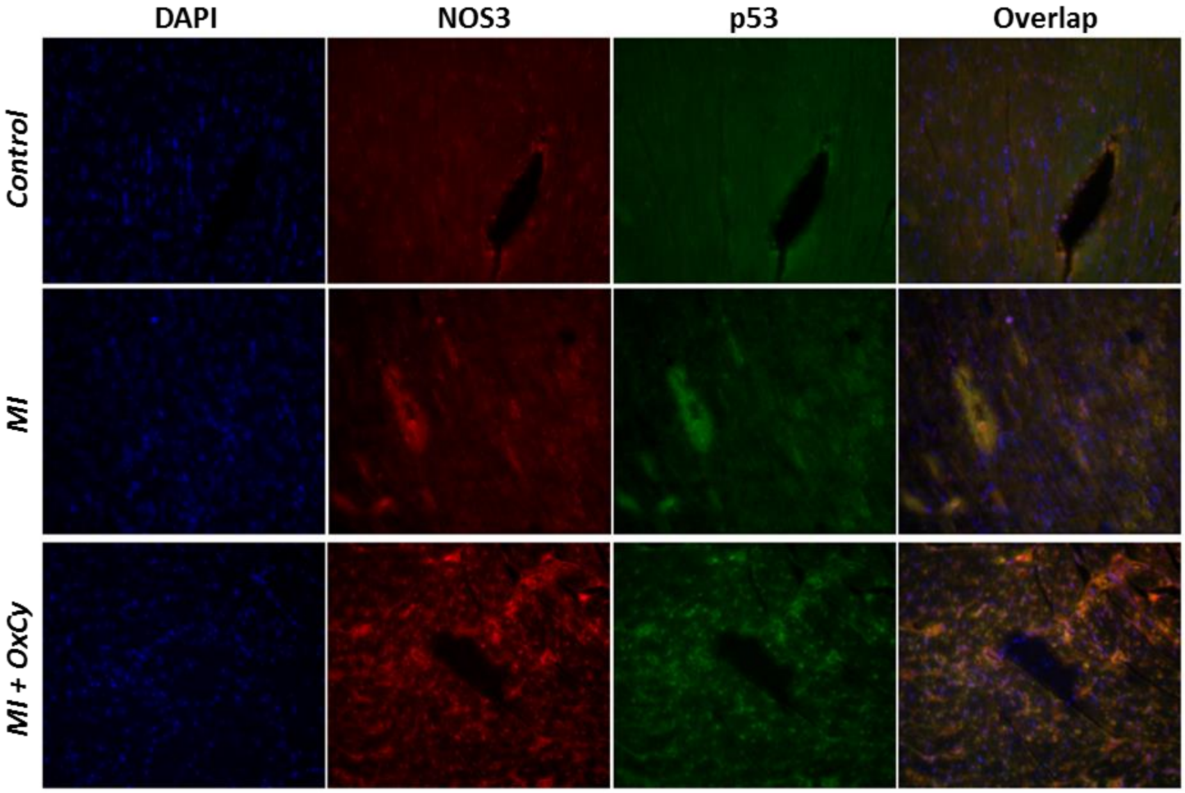

Figure S6. Co-localization of p53 and NOS3 proteins in the infarct heart tissue. Immunohistochemical staining of NOS3 and p53 were performed using NOS3 and p53 antibodies. The fluorescence microscopic images of DAPI (nuclear staining), p53 and NOS3 are superimposed to show their co-localization in the healthy (control), infarct (MI) and oxygen-treated (MI+OxCy) hearts.

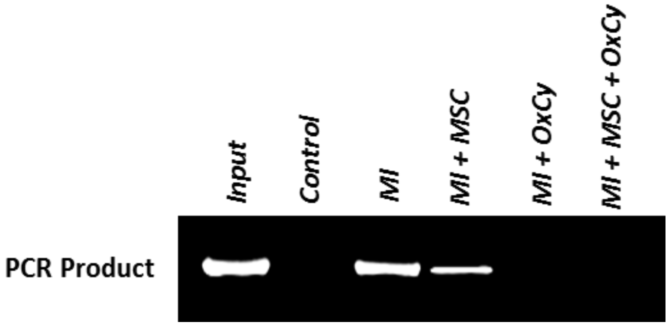

Figure S7. Binding of p53 protein on the BAX gene promoter. Chromatin immunoprecipitation (ChIP) was used to analyze the binding of p53 protein on the BAX gene promoter in the MI and MI+OxCy treated cardiac tissue. Results show that p53 does not bind to the BAX promoter in the healthy hearts, however strong p53 binding to the BAX promoter was observed in the MI heart tissue (lane 3). MI+SC hearts serve as controls (lane 4). p53 does not bind to BAX promoter in the MI+OxCy cardiac tissue (lane 5).

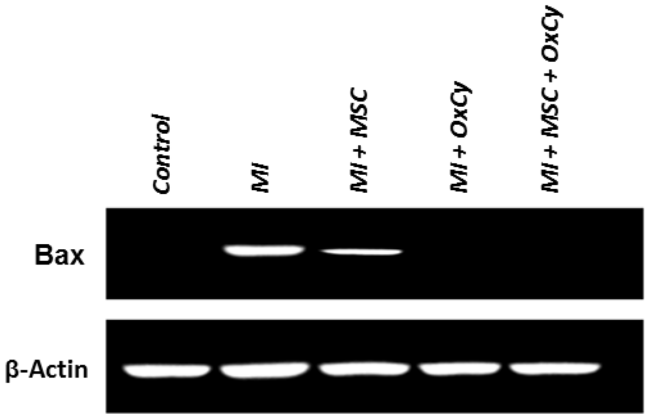

Figure S8. BAX mRNA level in the infarct heart tissue. BAX mRNA levels in MI and treated hearts were measured using RT-PCR. Results show that BAX mRNA is upregulated in the MI hearts. MI hearts treated with mesenchymal stem cell (MI+MSC), oxygenation (MI+OxCy), or combination (MI+MSC+OxCy) abolished BAX mRNA synthesis.

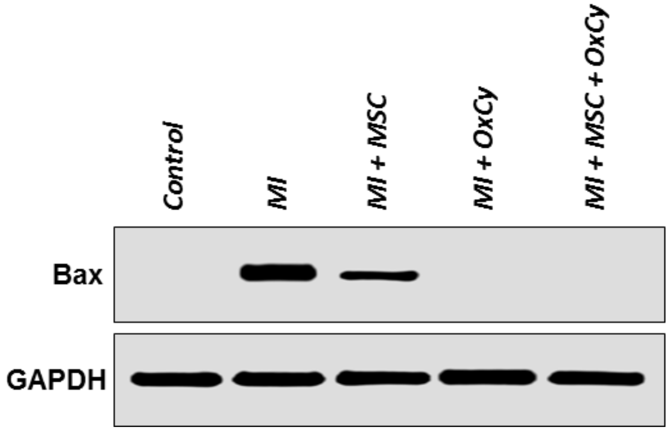

Figure S9. BAX protein level in the infarct heart tissue. BAX protein levels in MI and treated hearts were measured using western blotting. Results show that BAX protein is upregulated in the MI heart. MI hearts treated with mesenchymal stem cell (MI+MSC), oxygenation (MI+OxCy), or combination (MI+MSC+OxCy) abolished BAX protein.

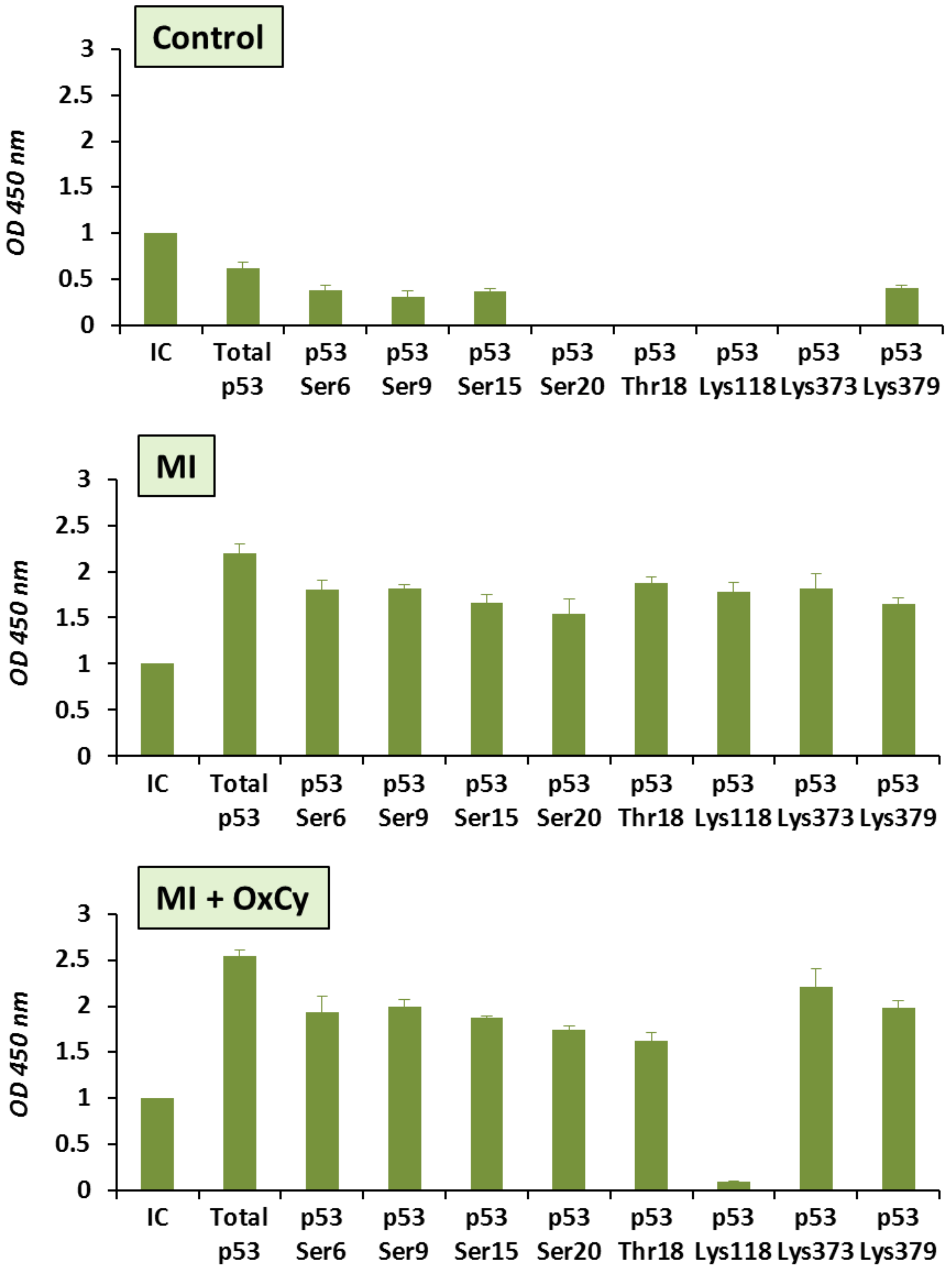

Figure S10. In vivo ELISA of p53 modifications. Phosphorylation and acetylation of p53 at the known sites were quantified in the healthy (control), MI and the MI+OxCy hearts. The results show significant increase in p53 phosphorylation of acetylation in the MI hearts in comparison to the control hearts. Interestingly the acetylation of p53Lys118 residue is significantly decreased upon oxygenation of the MI hearts. Data represent mean±SD of 7 independent measurements.

2. SUPPLEMENTAL EXPERIMENTAL PROCEDURES

Western blotting

Tissue homogenates were prepared from the anterior wall of the left ventricles of rats from all the four groups along with untreated control. After the treatment period, rats were anesthetized and sacrificed at 4 weeks of MI. Control rats (non I/R, n=4) were also used. The hearts were rapidly excised, rinsed in ice-cold PBS at pH 7.4 containing 500-U/ml heparin to remove red-blood cells and clots, frozen in liquid nitrogen and stored at -80°C until analysis. The proteins were isolated from tissue samples using Total Protein Extraction kit from Millipore. The tissue homogenate was incubated for 60 min on ice, followed by micro-centrifuging at 10,000× for 15 min at 4°C. Aliquots of 75 µg of protein from each sample were boiled in Laemmli buffer (Bio-Rad Laboratories, Hercules, CA) containing 1% 2-mercaptoethanol for 5 min. The protein was separated by SDS-PAGE, transferred to polyvinylidene difluoride (PVDF) membrane, and probed with primary antibodies for desired protein. The membranes were incubated overnight at 4oC with the primary antibodies, followed by incubation with horseradish peroxidase-conjugated secondary antibodies (Amersham Biosciences, Piscataway, NJ) for 1 h. The membranes were then developed using DAB as substrate (Vector laboratories).

Preparation of nuclear and cytoplasmic extracts

The tissue was spun at 2000 rpm for 10 min at 4°C and the pallet was collected. This pallet was suspended in 5x volume Buffer A (10 mM HEPES, pH7.9, 4°C, 1 ml; 1.5 mM $MgCl_2$, 150 µl; 10 mM KCl, 500 µl) raise to total volume of 100 ml, 4°C, ice min ~12 ml. Again the pallet was collected by spinning at 2000 RPM 10 min, 4°C. The palette was resuspended in 2x volume Buffer A ~5 ml and again homogenized using the homogenizer. The pellet was again collected by spinning at 2000 RPM 10 min, 4°C. From this point pellet and supernatant were treated separately.

A. Pellet consists of nuclei which were transferred to ultracentrifuge tubes and spun at 16,000 RPM at 4 degrees. The pellet was re-suspend in 0.6 ml buffer C (20 mM HEPES/TRIS 7.9 2 ml, 25% v/v glycerol 50 ml, 0.42 M NaCl 10.5 ml, 1.5 mM $MgCl_2$ 150 µl, 0.2 mM EDTA 40 µl, cocktail protease inhibitors 0.5 mM) raise total volume to 100 ml. The pallet was again homogenized and left to stir at 4°C for 30 minutes. Again the pellet was collected by spinning at 16000 RPM for 30 minutes. The pellet was incubated with buffer D (20 mM Hepes/Tris 7.9 2 ml, 20% v/v glycerol 40 ml, 0.1 M KCl 5 ml, 0.2 mM EDTA 40 µl, protease inhibitor 0.5 mM with total volume raised to 100 ml) for 5 hours. Finally the suspension was spun at 16000 RPM for 20 minutes and the supernatant was collected. The supernatant is the nuclear extract.

B. Supernatant is the cytoplasmic extract. To the cytoplasmic fraction 0.11 vol of buffer B ((0.3 M Hepes/tris 7.9 30 ml, 1.4 M KCl 70 ml, 0.03 M $MgCl_2$ 3 ml) and total volume raised to 100 ml) was added. The suspension was spun at 17000 RPM for 4 hours at 4°C and the supernatant was incubated with buffer D for 8 hours. Again the supernatant was collected after spinning the suspension at 17000 RPM for 4 hours at 4°C. The supernatant is the cytoplasmic fraction.

Immunoprecipitation

The proteins were isolated from tissue samples using Total Protein Extraction kit from Millipore. After cell lysis the extracted DNA was fragmented by passing the lysed suspension 5 to 10 times through a needle attached to a 1-ml syringe. The lysates were pre-cleared to help reduce non-specific binding of proteins to agarose or sepharose beads. Pre-clearing with an irrelevant antibody or serum will remove

proteins that bind immunoglobulins non-specifically. Thus to the lysate 50 µl of GAPDH (irrelevant) antibody of the same species (rabbit) was added for 3 hours to pre-clear the lysate. To this pre-cleared lysate antibody of NOS3 and p53 were added. Lysate was incubated with these antibodies overnight on ice and then 100 µl of bead slurry was added to the lysate. The bead and lysate mix was incubated for 4 hours at 4°C with gentle agitation. The mixture was spun at 14,000xg at 4°C for 10 minutes. The bead pellet was discarded and the supernatant was keep for immunoprecipitation. In order to increase the yield, the beads were washed 1 or 2 more times in lysis buffer, and the supernatants were collected. True blot anti body system from Affymetrix was used to get rid of heavy and light chain staining.

Annexin-V staining

Beckton Dickinson flow cytometer was used to detect the apoptotic cell surface shift of phosphatidylserine by the binding of fluorescein isothiocyanate (FITC) conjugated Annexin V to the outer membrane of intact cells. Floating cells were collected by centrifugation and these, as well as the attached cells were washed in PBS (Ca^{2+} or Mg^{2+} free)/0.1% EDTA. The attached cells were gently scraped off the dish and centrifuged. After centrifugation, the cells were treated with 500µl binding buffer (Abcam) then 5 µl of Annexin V-FITC and 5-µl propidium iodide was added. Cells were treated at room temperature for 5 minutes. These cells were then filtered through 70m mesh, to eliminate cell aggregates and analyzed by flow cytometry.

Protein ELISA

Sandwich ELISA (in vivo ELISA) from cell or tissue lysate was done as follows; The wells of a PVC microtiter plate were coated with 100 µl of 5 µg/ml of the desired antibody overnight at 4°C. After washing thrice with TBS buffer (0.05% Tween-20 in PBS), blocking was done using 5% skimmed milk in TBS for 2 hours at 4°C. After washing the wells thrice with TBS, 200 µg cell lysate in NP-40 buffer (normal/heat shocked) 11 v/v diluted in 5% skimmed milk in TBS was added to each well and incubated at 4°C for 2 hours. Subsequently 100 µl of desired polyclonal antibody was added to each well and incubated at 4°C for 2 hours. Again after three quick washes, 100 µl of HRP-conjugated desired secondary antibody was added to each well and kept at RT for another 2 hours. ELISA was developed for 30 minutes and O.D. was recorded at 450 nm on Microplate reader (Bio-Rad).

Induction of myocardial infarction

Male Fisher-344 rats (200-250 g) were used. Rats were randomly divided into four groups of 8 animals each: (1) MI group (serum-free medium-treated); (2) Ox group (MI with hyperbaric oxygen treatment); (3) MSC (MI with MSC transplantation); (4) MSC+Ox (MI with MSC and HBO treatment). Rats were anaesthetized with ketamine (50 mg/kg, i.p) and xylazine (5 mg/kg, i.p.) and maintained under anesthesia using isofluorane (1.5-2.0%) mixed with air. Myocardial infarction (MI) was created by ligating the left-anterior-descending (LAD) coronary artery for 1 h. After 1 h of ischemia, the ligation was released, flow was restored (reperfusion), and the chest cavity was closed. After ligation of the LAD coronary artery, successful infarction was confirmed, for all groups, by an ST elevation on the electrocardiograms. After reinstallation of spontaneous respiration, animals were extubated and allowed to recover from anesthesia. No mortality was observed in any of the groups at the end of the study. All the procedures were performed with the approval of the Institutional Animal Care and Use

Committee of The Ohio State University and conformed to the Guide for the Care and Use of Laboratory Animals (NIH Publication No. 86-23).

Protocol for oxygen-cycling

The rats were subjected to hyperoxygenation treatment by placing them inside a custom-built small-animal hyperbaric chamber (Polyfab; Boston Plastics Manufacturing, Wilmington, MA) connected to a compressed gas cylinder containing 100% oxygen. Oxygen (OxCy) was administered daily (100% O_2, 2 ATA, 90 min) starting 4 days after MI. OxCy was administered 5 days a week for 4 weeks. The animals were placed 1 per cage with up to 3 cages at a time in the chamber. After the chamber was closed, a fill-valve was slowly opened to allow the pressure within the chamber to reach 2 ATA (atmospheric pressure absolute) over 5-10 minutes. The valve was then closed, sealing the chamber and the rats kept in the chamber for 90 minutes under constant observation. After this period, a safety pressure-release valve was manually activated to slowly depressurize the unit to normal atmospheric pressure over 5-10 min.

Immunohistochemical staining of cardiac tissue for p53 and NOS3 expression

Hearts were fixed in formalin and embedded in paraffin. Five-micron sections were cut and preincubated in blocking buffer (5% nonfat dry milk on 0.1 mM PBS) for 45 minutes at 37°C, followed by overnight incubation with the primary antibodies anti-p53 (mouse monoclonal antibody for p53; sc-126; Santa Cruz Biotechnology) or anti-NOS3 (affinity purified rabbit polyclonal antibody for NOS3, sc-654; Santa Cruz Biotechnology) at 4°C. The sections were extensively rinsed in 0.05 mM PBS and incubated with peroxidase-conjugated secondary antibodies (1:100) for 2 hours at 25°C. After washing as above, sections were subjected for 5 minutes to freshly prepared diaminobenzidine that contained H_2O_2 (0.8%). The specimens were gently rinsed in 1× PBS, air-dried and mounting medium with DAPI (Vector labs, CA) was applied topically and slides were viewed under fluorescence microscope (Nikon TE 2000, Japan).
